# Identification and Evolutionary Analysis of Potential Candidate Genes in a Human Eating Disorder

**DOI:** 10.1155/2016/7281732

**Published:** 2016-03-21

**Authors:** Ubadah Sabbagh, Saman Mullegama, Gerald J. Wyckoff

**Affiliations:** Molecular Biology and Biochemistry, University of Missouri-Kansas City, 5007 Rockhill Road, Room 521, Spencer Chemistry Building, Kansas City, MO 64110, USA

## Abstract

The purpose of this study was to find genes linked with eating disorders and associated with both metabolic and neural systems. Our operating hypothesis was that there are genetic factors underlying some eating disorders resting in both those pathways. Specifically, we are interested in disorders that may rest in both sleep and metabolic function, generally called Night Eating Syndrome (NES). A meta-analysis of the Gene Expression Omnibus targeting the mammalian nervous system, sleep, and obesity studies was performed, yielding numerous genes of interest. Through a text-based analysis of the results, a number of potential candidate genes were identified. VGF, in particular, appeared to be relevant both to obesity and, broadly, to brain or neural development. VGF is a highly connected protein that interacts with numerous targets via proteolytically digested peptides. We examined VGF from an evolutionary perspective to determine whether other available evidence supported a role for the gene in human disease. We conclude that some of the already identified variants in VGF from human polymorphism studies may contribute to eating disorders and obesity. Our data suggest that there is enough evidence to warrant eGWAS and GWAS analysis of these genes in NES patients in a case-control study.

## 1. Introduction

Obesity is a markedly prevalent problem within the developed world, and eating disorders may be partly to blame for this. However, many patients, by default, receive a diagnosis of “eating disorder not otherwise specified” [1, 2]. Nonetheless, EDs have biochemical indicators, such as serotonin, norepinephrine, and dopamine, which can be detected [3].

Night Eating Syndrome (NES) is a disorder characterized by a patient eating at night, with full awareness, and the person may not be able to return to sleep unless they eat. More formal diagnoses include eating a certain percentage of calories (25% or more) after the last evening meal, or with multiple episodes per week of waking specifically to eat. Patients diagnosed with this disorder have a series of other problems (obesity, dental issues, etc.) that may revolve around the consumption of too many calories or the disruption of normal metabolism [4]. Dispute in the academic community centers around whether or not NES is characterized by disinhibition or other primarily neural issues, or metabolic/circadian issues that might be primarily physiological in nature [5]. We carried out our initial study assuming that both neural and metabolic factors may be playing a role in something that is a relatively common disease, affecting perhaps 1-2% of the general population and as many as 10% of obese individuals [4].

Deeper analyses of EDs in recent years have shown that there are genes that may play a role in ED. Notably, the TLQP-21 derivative of VGF gene has been linked with the prevention of obesity in diet-induced mice [6–8].

Neuropeptide precursor VGF is a secreted, proteolytically digested protein synthesized by neurons [9]. The gene and protein name is nonacronymic, despite potential action on vasopressin. Peptides derivative of VGF have been linked to synaptic plasticity, memory, circadian rhythm, depression, regulation of arginine vasopressin (AVP) secretion, and homeostasis [10–14]. VGF-derived peptide TLQP-21, its parent peptide TLQP-62, and the neuroendocrine regulatory protein, NERP-3, have been identified as bioactive with functions in regulating energy expenditure, memory, and homeostasis, respectively [6, 13, 15, 16]. However, the mechanisms of this activity remain largely unknown [16]. Other derivative peptides have also been identified as active (NERP-1, NERP-2, and NERP-4), but their functions have yet to be fully characterized [8, 9].

TLQP-21 appears to be further derived from the TLQP-62 peptide, an abundant peptide of VGF, and has been demonstrated to dose-dependently increase energy expenditure in rats through an intracerebroventricular treatment [6, 7]. It also increased uncoupled protein (UCP-1) in white adipose tissue. In the same study, TLQP-21 has also been shown to prevent the effects of high-fat diet in mice that were fed for 14 days [8, 17, 18]. Furthermore, in Siberian hamsters, a seasonal energy balance effect of TLQP-21 was observed, where intracerebroventricular infusions decreased food intake and body weight. This effect was ultimately attributed to a decrease in food intake per feeding session, rather than a reduction in energy expenditure [19].

NERP-3 has been identified in only one study as a biologically active peptide using a comprehensive peptidomic approach [11]. NERP-3 appeared to colocalize with AVP in the paraventricular nucleus and supraoptic nucleus of the hypothalamus. As the suprachiasmatic nucleus is responsible for the regulation of circadian rhythm, the distribution of NERP-3 there as illustrated in that study therefore suggests that the peptide might act as a mediator in the control of circadian rhythm, and VGF-deficient mice have been shown to have a shorter circadian time [19]. NERP-3 is also manifestly more conserved in sequence, in comparison to NERP-4, among mammalian species. NERP-4 has identical amino residues in humans, mice, and rats. It has only been found to be functional within the hypothalamus and pituitary. Its function, however, has not been characterized in any study [16].

The wide-ranging effects of the VGF-derived peptides determined in previous studies suggest strongly that VGF is a regulator of several processes involved in metabolism and therefore may be a good candidate gene to study to find potential up- and downstream genetic factors involved in metabolic disorders and more broadly eating disorders.

We examine VGF and three other genes identified in a broad screen of the Gene Expression Omnibus for potential involvement in Night Eating Syndrome. These genes were broadly selected because of their functions as detailed in Gene Ontology and their relationship to processes that might be important to NES specifically. The proteins coded for by these genes include CALR, MANF, and HTRA1.

In addition, examining overlap in significant data across our examined GEO datasets, we found several genes that were significant in two different datasets and broadly examined their identity and function.

## 2. Materials and Methods

### 2.1. Expression Analysis

The datasets for the analysis were collected from the Gene Expression Omnibus (GEO) [20]. The GEO website has a new feature named GEO2R that does some analysis of datasets [21]. The GEO2R does normalization of data with log⁡2 transformation along with Benjamini & Hochberg (false discovery rate) plus adding the annotation information. The GEO2R provides the R script of the online process that GEO2R performed for the particular dataset. GEO2R uses version 2.14.1 of R to perform the analysis. In order for the GEO2R to perform analysis, experimental groups must be assigned. Given our interest in the linkage between sleep and obesity, the datasets we were interested in (1) had to be in mammals, particularly humans or model organisms, (2) preferably should have the design of control versus experimental group or involve time course data allowing splitting into defined groups, and (3) should reflect differences in sleep habits; obesity or, more broadly, metabolism; and brain function and/or development. The following datasets from GEO were passed through the GEO2R online application: GSE3293, GSE2870, GSE2871, GSE96, GSE4692, GSE4697, GSE2392, GSE6514, GSE8700, GSE19185, GSE39375, and GSE929 (Table 1). Samples were assigned to the case or control group depending upon the experiment being examined. For the time course experiments involving sleep, disturbed sleep was compared to undisturbed sleep across all categories, which bins “disturbed” versus “undisturbed” sleep studies for comparison. For this time course, “undisturbed” sleep was used as a control. The GEO2R used the limma package from Bioconductor to display the *t*-test score, *p*-test score, and adjusted *p*-score. After each dataset was analyzed by GEO2R, the supplementary R script was copied and the *p* value was changed to the critical value of 0.001 and also set to output only values reflecting that *p* value. This change facilitated downstream analysis, while still allowing for significant corrected data to be seen. The output of the data was imported to Excel where the *Z*-score was calculated through the use of the inverse normal distribution (NORMSINV) function. All of the analyses from the GEO2R and the calculated *Z*-score were combined. Genes of interest were selected by using criterion of |*z* | ≥5.5, which is well above |*z* | ≈5 that corresponds to a Bonferroni-corrected *p* value of less than 0.05. We also subsequently examined any significant genes that overlapped between any of the datasets we examined.

### 2.2. Evolutionary Analysis

A sample of 55 VGF coding sequences were collected from the publicly available database NCBI (http://www.ncbi.nlm.nih.gov/), accessed on 06/22/14 for analysis. The sequences were exclusively from eutherian mammals of different clades (Table 2).

VGF mRNA coding nucleotide sequences were translated to amino acids, aligned using ClustalW [22], and adjusted manually. Phylogenetic reconstruction of the resulting 2028 bp alignment was conducted using MEGA version 6 [23]. Trees were constructed by maximum likelihood and Nearest-Neighbor Interchange (NNI) with 500-bootstrap replication.

The various missense mutations in human CALR, HTRA1, MANF, and VGF sequences from the results of the GEO meta-analysis discussed above were obtained from dbSNP [24] and screened for potential deleterious effects by two* in silico* methods: SIFT [25] and PolyPhen-2 [26]. SIFT scores substitutions numerically from 0 to 1. The amino acid substitution is predicted to be damaging if the score is ≤ 0.05 and otherwise is considered tolerated. The output of PolyPhen-2 is a prediction of either “probably damaging” (high confidence), “possibly damaging,” or “benign” accompanied by a score ranging from 0.0 (benign) to 1.0 (damaging). The sequences and structures sourced by PolyPhen-2 in our analysis are UniProtKB/UniRef100 Release 2011_12 (14 Dec. 2011) and PDB/DSSP Snapshot (03 Jan. 2012) (78304 structures), respectively.

### 2.3. Statistical Analysis

In order to evaluate evolutionary rates across species, the McDonald-Kreitman test [27] was used and assessed with Fisher's Exact Test. This test was conducted on CALR, HTRA1, MANF, and VGF selected from the results of the GEO meta-analysis discussed above. The goal was to compare the synonymous and nonsynonymous variation in each gene within humans and between humans and the house mouse.* Mus musculus* represents a good subject of comparison as most of the relevant expression studies conducted so far have been conducted on mice and rats as model organisms. The numbers of synonymous and nonsynonymous variations within humans were obtained by querying dbSNP for synonymous codons and missense mutations which have been validated by the 1000 Genomes Project [28]. The specific interest in SNPs obtained from the 1000 Genomes Project is to ensure conservative but accurate estimate representation of variation, as information referenced from the project is from verified human genomic data. To obtain the same information for comparison between humans and mice, we aligned the protein coding nucleotide sequence of both species using ClustalW [22] in the MEGA6 package [23] and evaluated the number of synonymous and nonsynonymous substitutions. *p* values below 0.05 were considered significant.

## 3. Results

### 3.1. Expression Analysis

The analysis of all datasets resulted in 1,052 genes that are significant and possible candidates for further studies. From the datasets, the following number of genes was significant: GSE3293 (172 genes), GSE2870 (164 genes), GSE2871 (40 genes), GSE96 (64 genes), GSE4692 (35 genes), GSE4697 (107 genes), GSE2392 (25 genes), GSE6514 (200 genes), GSE8700 (120 genes), GSE19185 (35 genes), GSE39375 (37 genes), and GSE929 (52 genes). The complete list of these genes is available upon request. Many of these genes had little if any annotation. Following up on all data including from the GEO annotation of the original experiments, GeneCards data, and GO functions, a set of potential candidates with interesting functionality with some identification in both neural development and sleep and obesity or eating disorders was assembled. From this sample of significant data, 38 were selected for further, more in-depth literature analysis (Table 3(a)). Many of these genes appeared in dataset GSE6514, in part because the genes in this set overall had better available literature; thus, despite the wide representation of genes across datasets, this set is overrepresented in the genes from the final literature review list in Table 3(a). We undertook a review of the literature of these selected genes, paying particular attention to the definition of NES and any attendant functions. While many of these genes could potentially be involved in the link between sleep and obesity that we sought to define, the most promising of the 38 genes examined is the gene VGF. Three other genes also stood out because of their Gene Ontology [29] functionalities or other literature. In addition, we examined all data across sets to see if any genes were significantly different in more than one dataset. Our results (Table 3(b)) show that 6 genes met this stringent criterion: they had varying levels of functional annotation available, described below.

Calreticulin (CALR) is a calcium binding protein that is likely to have a role in transcriptional regulation. Importantly, the protein has been shown to inhibit the binding of androgen receptor* in vivo*, as well as having a role in retinoic acid-induced neuronal differentiation [30]. CALR binds to the amino acid sequence motif, KXGFFKR. This amino acid motif is found in alpha-integrin as a cytoplasmic domain. The protein may inhibit commitment to adipocyte differentiation and has been found to be a candidate gene involved in sleep in* Drosophila* [31].

Mesencephalic astrocyte-derived neurotrophic factor (MANF) has been found to be important in the survival of dopaminergic neurons. Importantly, this is a highly conserved protein and recent studies suggest that it may be a candidate for modification to slow the progression of Parkinson's Disease. While it has a role in regulation of genes during expression, it has a broader pattern of expression in adult animals studied including within adipose tissue and the spleen and seems to protect against ischemic damage in the heart and brain [32]. It is upregulated in endurance training in rats [33].

HtrA serine peptidase 1 (HTRA1) is a member of the trypsin family of serine proteases and is likely a regulator of cell growth. Associated with autosomal-dominant cerebral small-vessel disease and age-related macular degeneration (and variations within the promoter region of the gene have been found to be causal), the protein regulates the availability of insulin-like growth factors by cleaving IGF binding proteins [34–36]. Perhaps, more interestingly, HTRA1 has been found to play a role in gingivitis and aggressive periodontitis [37]. This is particularly interesting as there is a link between NES and tooth loss that is, as yet, not sufficiently explained but has been hypothesized to be due to lower salivary production at night.

VGF is associated with such biological processes such as glucose homeostasis, insulin secretion, and response to dietary excess. A recent study has suggested that TLQP-21, a VGF-derived peptide, is involved in increased energy expenditure and prevents the early phase of diet-induced obesity. We felt that this was important, as the VGF gene itself was found to be significant in a study related to sleep and brain development, but the available literature linked VGF with homeostatic processes, insulin secretion, and other metabolic functions that could be associated with obesity. Therefore, the fact that VGF was isolated from one screen but found to be involved in processes outside those relative to that screen was considered when we moved forward with further analysis.

The genes that showed overlap across two GEO datasets were relatively varied in their function. Of these, insulin-like growth factor binding protein 2 (IGFBP2) was potentially the most interesting, as its regulation of insulin appears to have implications both in epithelial growth and differentiation in cancer [38] and in protection from obesity and insulin resistance [39]. Due to its function and clear implications of the protein in obesity within a model organism, we would suspect that it is a good potential candidate gene for Night Eating Syndrome.

The remaining genes that were found to be significant do not appear, from our review of the literature, to be as intricately involved in both brain and metabolic processes as these genes. This, of course, does not exclude them from being potentially significant drivers of NES and we feel that they should be included in future analyses, perhaps focusing on circadian rhythms [40]. However, for the purposes of this analysis, we chose to focus on these genes as the most suitable potential candidates for future research on biomarkers associated with eating disorders in part because there is already a foundation of research supporting the hypothesis that they play a role in processes important to Night Eating Syndrome, but they have not necessarily been tied directly to night eating and little is known of their evolutionary history. We therefore proceeded with an evolutionary analysis of these genes. While this list could be seen as arbitrary, our goal was to show that some subsets of the identified genes were potential candidate genes for involvement in NES, not to be exhaustive in analyses of these lists.

As noted, VGF appears to act on downstream targets via peptides. We analyzed the evolution of these peptides as well as the variation of these peptides within humans and between humans and other mammalian species. We further investigated CALR, MANF, and HTRA1, examining both SNPs and their evolution utilizing the McDonald-Kreitman test (Table 5) [27].

### 3.2. Evolutionary Analysis of Positive Selection

The PAML-4 [41, 42] package offers a program CODEML which uses codon substitution models to perform maximum likelihood analyses of coding sequences. Several of these models available in the program were used to estimate different *dN*/*dS* ratios (*ω* parameter). The opposing models (neutral) were also conducted and compared using likelihood ratio tests (in an effort to avoid false positives). The F3x4 estimation of codon frequencies was used. The tests run were the one-ratio (M0), neutral (M1a), selection (M2a), beta (M7), and beta&*ω* (M8) models. M7 and M8 are opposing models, where M7 does not allow for positive selection and assumes beta-distribution for *ω* ≤ 1 and M8 allows for positive selection (*ω* > 1). M8 identified some sites as possible regions of positive selection based on the Bayes empirical Bayes (BEB) approach. A likelihood ratio test (LRT) (Table 4) was applied to evaluate the significance of the result using the formula 2Δln⁡L to compare the M8 model to the null M7 model with d.f. = 2. The LRT did not reach statistical significance, as the calculated LRT value 5.573 fell slightly short of the chi-squared value 5.991. Further, M1 could not be rejected in favor of M2, which allows for positive selection, as the LRT yielded a value of 0. Therefore, overall positive selection of the gene could not be inferred from these tests.

**Figure 1:**
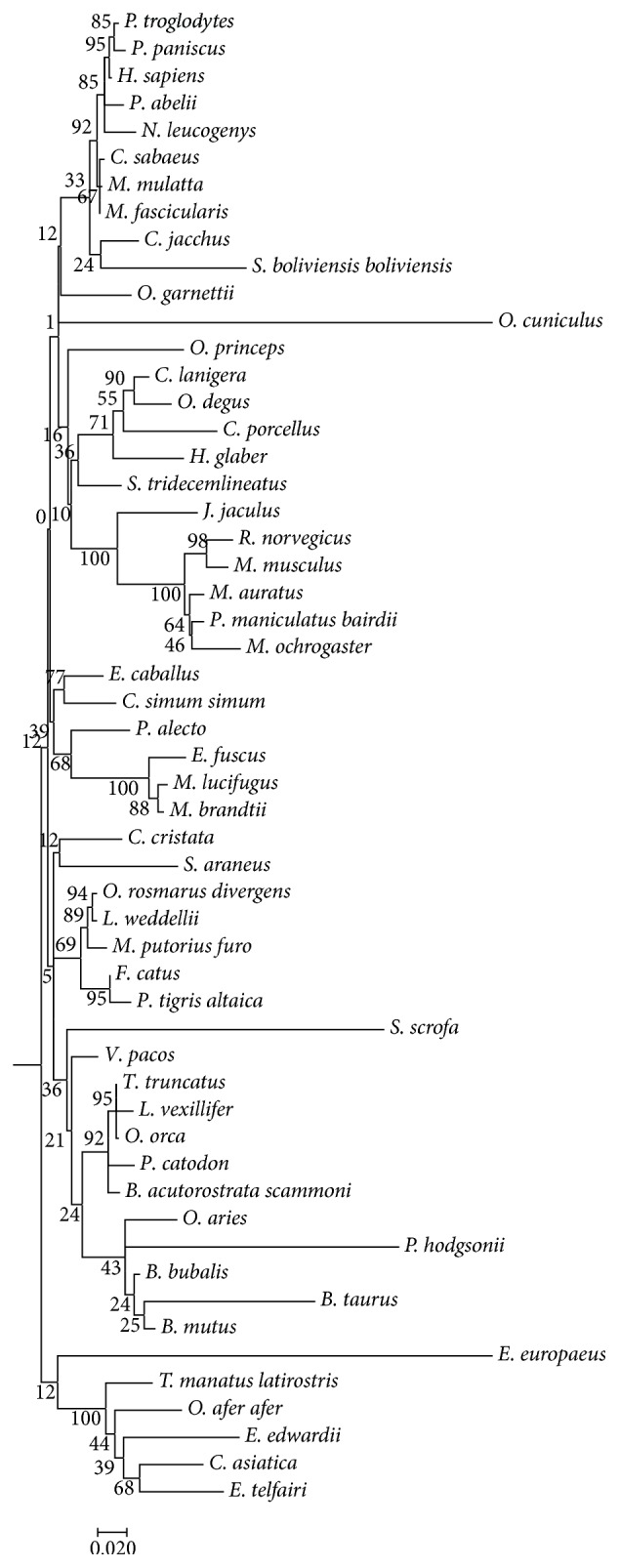
A phylogenetic tree was constructed by maximum likelihood and Nearest-Neighbor Interchange (NNI) with 500-bootstrap replication. The numbers at the nodes are indications of bootstrap reliability, showing percentages of times the node was replicated within the bootstrap trials. Most of these clusters are calculated to be reliable at >70%. We did not collapse weak nodes, but they are noted on the tree; trees with nodes that read 0 are very weak and were rounded to 0% representation within the bootstrap replicates. Generally, the higher confidence nodes reflect more divergence as well. There is excellent support for the generally accepted primate node.

Though the overall negative test does not allow inference of selection acting on the entire gene, multiple sites were calculated to have a *dN*/*dS* ratio greater than 1 by using both methods of naive empirical Bayes (NEB) and the Bayes empirical Bayes (BEB). The specific sites predicted to be under positive selection are presented in Table 4. As shown on the graph in Figure 1, these sites largely fall at positions within and downstream of identified peptides NERP-4 and TLQP-21. That these sites clustered near functionally important peptides strongly suggests that changes within these peptides might have functional significance, warranting further investigation. Even when factoring in the calculated standard error, some of the ratios were estimated to be comfortably above *ω* = 1. Results of the NEB estimation are reported for the purpose of within-protein comparison.

### 3.3. Phylogenetic Analysis

The evolutionary history was inferred by using the maximum likelihood method based on the JTT matrix-based model [43]. The tree with the highest log-likelihood (−10181.7394) is shown. All positions with less than 35% site coverage were eliminated. That is, fewer than 65% alignment gaps, missing data, and ambiguous bases were allowed at any position. There were a total of 617 positions in the final dataset. Evolutionary analyses were conducted in MEGA6 [23]. The phylogenetic reconstruction looked relatively typical for a mammalian gene; however, certain lineages were much more highly derived. One working hypothesis at this point is that shifts in diet and, importantly, the timing of eating may have contributed to the evolutionary pattern shown (Figure 1).

### 3.4. Variation Analysis in VGF-Derived Peptides

**Figure 2:**
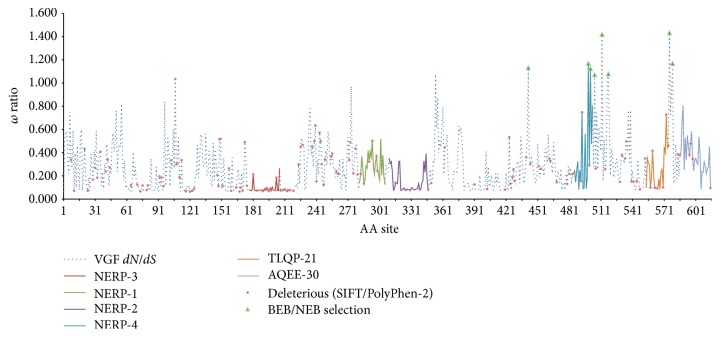
Mutations and peptide identities in VGF along a running *dN*/*dS* calculation for the 615-amino acid protein. Mutations are noted in red, with the exception that mutations in a region *dN*/*dS* > 1.0 are green. Peptide identities are represented in color on the graph and their identities are noted at the bottom of the figure.

Each currently identified peptide derivative of VGF, with even a modest amount of research available, was marked and mapped onto the amino acid sequence of the gene. Using publicly available resources, ENSEMBL [44] (http://www.ensembl.org/) and dbSNP [24] (http://www.ncbi.nlm.nih.gov/SNP/), we determined which currently existing variants are located within the regions of interest (Figure 2). This analysis was conducted strictly on human sequences, which have the most available variation data. We then ran several variation analysis tools to predict whether such mutations in sequence would be tolerated, theoretically, or damaging to function. In addition, we used the CODEML program provided in the PAML package to determine whether any part of the protein was, in theory, rapidly evolving or whether any amino acid sites were under selection.

**Figure 3:**
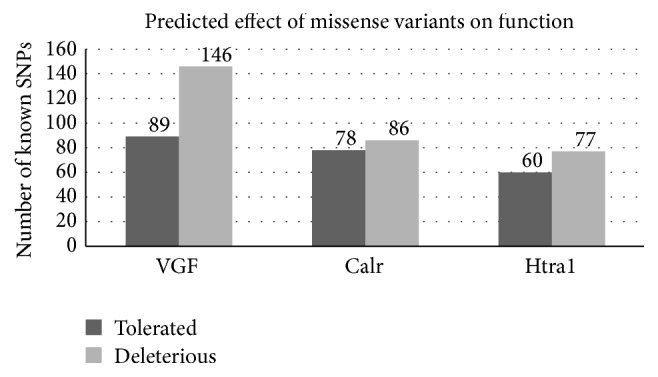
How nonsynonymous mutations are tolerated within VGF, CALR, and HTRA1 proteins. Predictions of functional consequences are determined by SIFT and PolyPhen-2. Darker regions are predicted to be “tolerated” or “benign” by both SIFT and PolyPhen-2, respectively. Bars with a lighter shade depict those mutations which are predicted to be “deleterious” and “probably damaging,” with the exclusion of “probably damaging” (lower confidence score) assessments from PolyPhen-2.

We therefore created a spreadsheet tool in which all coding variations in VGF can be examined. Several SNPs that were not well tolerated were found and plotted along a running *dN*/*dS* calculation for each amino acid (calculated from PAML). In general, VGF peptides were more conservative than the gene average, as evidenced by both the absence of significance when testing for overall positive selection and the calculated *ω* ratio (*dN*/*dS*) for the protein in its entirety. VGF-derived peptides of interest have been mapped onto the protein at their appropriate sites, allowing the exploration of mutations within specifically relevant regions. The least conserved portion of the protein was between NERP-4 and TLQP-21 peptides. The spreadsheet is available upon request from the corresponding author. Notably, using two common tools to determine how well mutations may be tolerated, we found that a large percentage of mutations were likely poorly tolerated or could contribute to protein dysfunction. As compared to CALR and HTRA1, VGF has a notably larger number of known variants in humans that are predicted to be detrimental to function, further establishing it as a more likely candidate to investigate (Figure 3).

Again, we saw that VGF had the lowest percentage of SNPs predicted to be tolerated according to analysis with both PolyPhen-2 and SIFT. MANF could not be examined due to the very low number of polymorphisms. All proteins were significant under the McDonald-Kreitman test, but this may be due to the presence of rare mutations within the 1000-genome polymorphism set examined [28] and MANF has such a low number of variants that the results must be considered with some suspicion. Regardless, the significant difference suggests that evolution has acted in a different fashion within humans than it has in the time frame between the divergence of mice and that of humans. While this is, in itself, not unusual, in all cases, the most parsimonious explanation was excess of nonsynonymous variation within humans.

## 4. Discussion

It is evident that VGF is a precursor to peptides with multiple functions and significant pathophysiological roles. The current body of research on this protein leaves many of these functions to be determined. Our aim was to determine whether there were potential candidate genes for NES within the human genome, and we believe we have established the notion that VGF appears to be a one such candidate. It has well conserved peptides that are known to function through downstream targets to affect metabolism and regulation of neurotransmitters [8, 10, 13, 45]. Its key regulatory position may provide it with a role as a modulator of metabolism through several mechanisms. For example, at least two possible candidates have been identified as receptors for the TLQP-21 peptide [15, 45]. This investigation is the first of its kind with respect to an evolutionary analysis of VGF, and, from that perspective, the sequence conservation within the peptides causes the mutations predicted to be damaging, or poorly tolerated, found within these peptides of potential clinical interest as either biomarkers or therapeutics (or both). The difference in evolutionary rate between different peptides derived from the protein is also an interesting facet and warrants further examination.

We believe that a next step approach may involve using VGF as a candidate gene in screens of obese versus nonobese humans in a case-control study focusing on both coding region SNPs and expression changes of VGF and downstream genes. Further examination of CALR1 and HTRA1 may also be warranted given the significant McDonald-Kreitman tests for both. We believe that examination of IGFBP2 is warranted given the function of the protein in model organism studies as well as the fact that it appeared as significant in two distinct GEO screens.

While any of the 1,052 significantly different genes identified have the potential to be candidates for NES (see Supplement 1 in Supplementary Material available online at http://dx.doi.org/10.1155/2016/7281732), we suggest that both the identification and analysis of the genes in this study, which indicate an ample number of genes that intricately link neural development and sleep behavior with eating disorders, warrant a rigorous case-control study of NES patients versus healthy weight controls, using more extensive genome-wide analyses. While NES is almost certainly a complex, multigenic disease (as are most other EDs), this data suggests that there might be genes with sufficient causal action to be pulled out in even a small-scale study focused on expression changes in an eGWAS screen or on associated SNPs using a GWAS screen. It is also possible that a study of obese, non-night eating patients versus obese NES patients may prove useful as our data suggest that they may not entirely overlap in genetic etiology. However, this would likely be a more problematic study to construct as ruling out night eating habits may prove to be rather difficult. Prior to this study, the impetus for performing any such analyses was not clear; we therefore believe that this study marks a significant advance in this area. While we were not exhaustive in our examination of the evolution of all 1,052 identified candidates, we feel that a next logical step involves examining how many of the genes are under circadian control and how many have excess of poorly tolerated SNPs relative to background frequency. For example, we know that VGF appears to be under circadian control in the suprachiasmatic nucleus (SCN) [40], and this pattern may hold for other genes in our dataset. These examinations could further expand the list of good potential candidate genes involved in NES, as well as potentially other diseases.

We also hypothesize that some of the missense mutations found in the variation screens conducted in this analysis are in fact involved in the modulation of metabolism and therefore likely affect metabolism and, potentially, weight. As these mutations are typically overlooked and used in control populations, we caution that experimental studies should be conducted with tighter control populations than may normally be considered in the study of these proteins. This is particularly notable in VGF, which appears to have a preponderance of mutations predicted to be poorly tolerated relative to other genes examined in this study (though this excess of poorly tolerated variation is only marginally significant compared to other proteins examined). Particularly, for studies of diseases which are as common as eating disorders, it may be especially necessary to examine healthy weight and behavior populations for establishing which variations are basal and which may be associated with any eating disorder. This may be particularly problematic for any disease associated with obesity, which has generally high prevalence worldwide [46].

## Supplementary Material

The Supplementary Material made available online is a list of 1,052 genes that resulted from all datasets analyzed in this study. These genes are significant and should be considered as possible candidates for further investigation, however, many of these genes had little if any annotation.

## Figures and Tables

**Table 1:** The GEO datasets analyzed in this study listed by accession, with the purpose of the experiment listed as well as the species examined and the number of samples.

GEO acc. ID	Species	Title/function	# of samples
GSE3293	*Mus musculus*	Leptin Regulated Mouse Gallbladder Genes	8
GSE2870	*Rattus norvegicus*	Ogle-5P01NS037520-05/chronic stress	18
GSE2871	*Rattus norvegicus*	giza-affy-rat-84719/brain injury	47
GSE96	*Homo sapiens*	Large-scale analysis of the human transcriptome	85
GSE4692	*Mus musculus*	Diet-induced obesity	6
GSE4697	*Mus musculus*	High-fat diet	8
GSE2392	*Mus musculus/Rattus norvegicus*	Brain injury	61
GSE6514	*Mus musculus*	Spontaneous sleep and prolonged wakefulness time course	90
GSE8700	*Rattus norvegicus*	Epididymal fat tissues of diet-induced obese rats	15
GSE19185	*Mus musculus*	Leptin treated ob/ob mice	8
GSE39375	*Mus musculus*	Obesity and fasting	10
GSE929	*Mus musculus*	Developing cortex	12

**Table 2:** This table shows the mammalian species examined in this study, along with the “accession.version” number of the VGF sequence analyzed.

Organism	Reference sequence
*Pongo abelii*	XM_002817792.1
*Chlorocebus sabaeus*	XM_008018472.1
*Macaca mulatta*	XM_001114019.1
*Pan troglodytes*	XM_003949223.1
*Callithrix jacchus*	XM_002743967.2
*Odobenus rosmarus*	XM_004399054.1
*Macaca fascicularis*	XM_005549512.1
*Leptonychotes weddellii*	XM_006733716.1
*Otolemur garnettii*	XM_003794166.1
*Mustela putorius*	XM_004761584.1
*Spermophilus tridecemlineatus*	XM_005328486.1
*Condylura cristata*	XM_004688258.1
*Chinchilla lanigera*	XM_005396865.1
*Rattus norvegicus*	NM_030997.1
*Trichechus manatus*	XM_004386747.1
*Equus caballus*	XM_001916011.3
*Heterocephalus glaber*	XM_004840148.1
*Pteropus alecto*	XM_006918591.1
*Tursiops truncatus*	XM_004312315.1
*Orcinus orca*	XM_004268845.1
*Ceratotherium simum*	XM_004442177.1
*Vicugna pacos*	XM_006201407.1
*Nomascus leucogenys*	XM_003276606.1
*Peromyscus maniculatus*	XM_006971252.1
*Mesocricetus auratus*	XM_005080400.1
*Sorex araneus*	XM_004620862.1
*Oryctolagus cuniculus*	XM_008250723.1
*Bos taurus*	XM_870373.5
*Octodon degus*	XM_004630477.1
*Lipotes vexillifer*	XM_007472135.1
*Saimiri boliviensis*	XM_003934284.1
*Myotis lucifugus*	XM_006106213.1
*Eptesicus fuscus*	XM_008141437.1
*Microtus ochrogaster*	XM_005344565.1
*Orycteropus afer*	XM_007939997.1
*Mus musculus*	XM_006504434.1
*Jaculus jaculus*	XM_004666201.1
*Ochotona princeps*	XM_004586958.1
*Chrysochloris asiatica*	XM_006859658.1
*Echinops telfairi*	XM_004705611.1
*Panthera tigris*	XM_007074009.1
*Homo sapiens*	NM_003378.3
*Physeter catodon*	XM_007126398.1
*Bubalus bubalis*	XM_006047303.1
*Elephantulus edwardii*	XM_006889712.1
*Balaenoptera acutorostrata*	XM_007187021.1
*Myotis brandtii*	XM_005879163.1
*Felis catus*	XM_004001431.1
*Cavia porcellus*	XM_003470139.2
*Pan paniscus*	XM_003807207.1
*Bos mutus*	XM_005892862.1
*Erinaceus europaeus*	XM_007517981.1
*Pantholops hodgsonii*	XM_005955976.1
*Ovis aries*	XM_004021273.1
*Sus scrofa*	XM_005658608.1

**(a):** 

Dataset (GEO)	ID	*p* value (adjusted)	Gene symbol	*Z*-score	Function (GO)
GSE6514	1423795_at	3.68*E* − 13	Sfpq	−8.79	DNA binding
GSE6514	1416332_at	2.76*E* − 10	Cirbp	−7.93	RNA binding
GSE6514	1442051_at	1.38*E* − 09	Hist2h3c1	−7.68	Negative regulation of transcription from RNA polymerase II promoter
GSE6514	1422660_at	1.46*E* − 09	Rbm3	−7.63	RNA binding
GSE6514	1452091_a_at	2.33*E* − 09	Rbm28	−7.54	RNA binding
GSE6514	1435854_at	5.53*E* − 09	Opalin	−7.41	Molecular function
GSE6514	1427464_s_at	6.11*E* − 09	Hspa5	−7.37	ATP binding
GSE6514	1425993_a_at	1.50*E* − 08	Hsph1	−7.23	ATP binding
GSE6514	1424638_at	3.36*E* − 08	Cdkn1a	−7.11	Cyclin-dependent protein kinase
GSE6514	1454725_at	1.42*E* − 07	Tra2a	−6.89	RNA binding
GSE6514	1417574_at	1.61*E* − 07	Cxcl12	−6.86	Growth factor activity
GSE6514	1416749_at	1.66*E* − 07	Htra1	−6.84	Insulin-like growth factor binding
GSE6514	1436094_at	3.05*E* − 07	Vgf	−6.74	Neuropeptide hormone activity
GSE6514	1420093_s_at	3.13*E* − 07	Hnrpdl	−6.73	DNA binding
GSE6514	1451566_at	3.93*E* − 07	Zfp810	−6.68	Metal ion binding
GSE6514	1416354_at	8.52*E* − 07	Rbmx	−6.56	RNA binding
GSE6514	1448654_at	9.60*E* − 07	Mtch2	−6.53	Transport
GSE6514	1439630_x_at	1.12*E* − 06	Sbsn	−6.50	Molecular function
GSE6514	1423796_at	1.23*E* − 06	Sfpq	−6.48	DNA binding
GSE6514	1441075_at	1.28*E* − 06	Nostrin	−6.47	DNA binding
GSE6514	1454014_a_at	1.94*E* − 06	Mkks	−6.40	ATP binding
GSE6514	1429862_at	2.03*E* − 06	Pla2g4e	−6.38	Phospholipase A2 activity
GSE6514	1428470_at	2.88*E* − 06	Exoc2	−6.32	Ral GTPase binding
GSE6514	1426722_at	3.53*E* − 06	Slc38a2	−6.28	Amino acid transmembrane transporter activity
GSE6514	1448454_at	4.83*E* − 06	Srsf6	−6.22	Negative regulation of mRNA splicing
GSE6514	1417303_at	6.37*E* − 06	Mvd	−6.17	ATP binding
GSE6514	1460645_at	8.39*E* − 06	Chordc1	−6.12	Hsp90 protein binding
GSE6514	1451047_at	9.49*E* − 06	Itm2a	−6.09	Integral membrane protein
GSE6514	1452318_a_at	9.49*E* − 06	Hspa1b	−6.09	NF-kappaB binding
GSE2871	AF020212_s_at	0.0000095	Dnm1l	−6.32	Apoptosis, inferred
GSE6514	1417606_a_at	1.00*E* − 05	Calr	−6.08	Androgen receptor binding
GSE6514	1452170_at	1.05*E* − 05	Chpf2	−6.06	Molecular function
GSE6514	1428112_at	1.08*E* − 05	Manf	−6.06	Growth factor activity
GSE6514	1435158_at	1.14*E* − 05	Rbm12b1	−6.04	RNA binding
GSE2870	1369751_at	0.00002325	TRHR thyrotropin releasing hormone receptor	−6.17	Thyrotropin releasing hormone receptor, GPCR signaling pathway
GSE2871	rc_AI172162_at	0.0000257	Psmb4	−6.01	Negative regulation of inflammatory response to antigenic stimulus
GSE2870	1367851_at	0.00005068	Prostaglandin D2 synthase	−6.00	Regulation of circadian cycle
GSE2392	1427660_x_at	0.00038711	IGK-V28	−6.04	Response to lipopolysaccharide

**(b):** 

Set 1	ID (set 1)	*p* value (adjusted), set 1	Gene symbol	Set 2	ID (set 2)	*p* value (adjusted), set 2	Broad function
GSE96	35670_at	3.11*E* − 03	Atp1a3	GSE2871	D00189_at	7.46*E* − 04	Dystonia in mice

GSE6514	1438635_x_at	3.70*E* − 04	B930041F14Rik	GSE19185	ILMN_1250201	1.79*E* − 03	Channel activity

GSE2871	AF055884_s_at	7.46*E* − 04	Deaf1	GSE6514	1448446_at	6.95*E* − 03	Zinc finger transcriptional regulator, SPN

GSE96	40422_at	2.03*E* − 03	IGFBP2	GSE19185	ILMN_2930897	7.56*E* − 03	Insulin-like growth factor binding protein

GSE6514	1422660_at	1.46*E* − 09	Rbm3	GSE19185	ILMN_1234453	7.50*E* − 03	RNA binding

GSE96	39756_g_at	5.81*E* − 03	Xbp1	GSE6514	1437223_s_at	4.75*E* − 05	MHC class 2 regulation

**Table 4:** Tests of overall positive selection of VGF protein using CODEML of PAML.

Model	ln L^a^	Parameter estimates	−2Δln L^b^	Positively selected sites
M0 (one-ratio)	−22230.135	*ω* = 0.240		

M3 (discrete)	−21925.088	*p*0 = 0.308; *ω*0 = 0.031 *p*1 = 0.580; *ω*1 = 0.272 *p*2 = 0.112; *ω*2 = 0.763	n.s.^e^	

M1a (neutral)	−22088.358	*p*0 = 0.870; *ω*0 = 0.184 *p*1 = 0.131; *ω*1= 1.000		

M2a (selection)	−22088.358	*p*0 = 0.869; *ω*0 = 0.184 *p*1 = 0.089; *ω*1 = 1.000 *p*2 = 0.042; *ω*2 = 1.000	n.s.	

M7 (beta)	−22007.790	*p* = 0.843, *q* = 2.462		

M8 (beta&*ω* > 1)	−22005.004	*p*0 = 0.976; *p* = 0.955 *q* = 3.090; *p*1 = 0.024; *w* = 1.054	n.s.	499, 501, 512, 576, and 579^c^; 442, 505, and 518^d^

^a^log-likelihood; ^b^likelihood ratio test (LRT) for detection of positive selection; ^c^sites inferred to be under positive selection pressure posterior probability by BEB method; ^d^sites inferred to be under positive selection pressure posterior probability by NEB method; ^e^not significant.

**Table 5:** Evaluation of McDonald-Kreitman 2 × 2 contingency table by Fisher's Exact Test (FET).

		Nonsynonymous	Synonymous^c^	Total
VGF				
*p* = 0.0011	Within species variation^a^	27	16	43
	Fixed species differences^b^	79	146	225
	Total	106	162	268

Calr				
*p* < 0.0001	Within species variation^a^	30	24	54
	Fixed species differences^b^	5	39	44
	Total	35	63	98

Htra1				
*p* = 0.0016	Within species variation^a^	19	22	41
	Fixed species differences^b^	45	163	208
	Total	64	185	249

Manf				
*p* = 0.0002	Within species variation^a^	7	2	9
	Fixed species differences^b^	5	39	44
	Total	12	41	53

^a^All known mutations within humans; ^b^differences when comparing human gene to mouse; ^c^only those polymorphisms identified by dbSNP as either synonymous or missense (nonsynonymous) are considered in this table. *p* values less than 0.05 are considered significant.
