# Supplementary material for: Identification and Evolutionary Analysis of Potential Candidate Genes in a Human Eating Disorder
Source: Biomed Res Int. 2016 Mar 21;2016:7281732. doi: 10.1155/2016/7281732 (PMC4819096; doi:10.1155/2016/7281732)
Supplement: Supplementary file 1 — The Supplementary Material made available online is a list of 1,052 genes that resulted from all datasets analyzed in this study. These genes are significant and should be considered as possible candidates for further investigation, however, many of these genes had little if any annotation. [file 7281732.f1.pdf]

GSE set #	ID	adj.P.Val	P.Value	t	Gene.Symbol
2871	AF020212_	9.5E-06	1.33E-10	8.235159	Dnm1l
2871	rc_AI63951	9.5E-06	2.23E-10	8.081836	
2871	rc_AI1721€	2.57E-05	9.06E-10	-7.67066	Psmb4
2871	AFFX-BioB-	0.000026	1.22E-09	-7.58256	
2871	rc_AA8008	2.98E-05	1.75E-09	-7.47808	Prickle2
2871	rc_AI6392!	4.69E-05	3.31E-09	7.292805	Dock8
2871	rc_AA8595	5.64E-05	4.65E-09	-7.19464	Arhgef7
2871	X93352_at	7.52E-05	7.08E-09	-7.07244	Rpl10a
2871	rc_AA8756	7.69E-05	8.14E-09	-7.03201	Ina
2871	M33025_s_	0.000374	4.40E-08	-6.54413	Ptms
2871	rc_AI1450	0.00045	6.22E-08	-6.44412	Mapk8ip2
2871	rc_AA7997	0.00045	6.38E-08	-6.43674	Polr2i
2871	rc_AA9247	0.00045	6.89E-08	-6.41487	Mt3
2871	rc_AI1788	0.000505	9.61E-08	6.318706	Eif4ebp1
2871	rc_AA8586	0.000505	1.04E-07	-6.29545	Lztr1
2871	rc_AI2285	0.000505	1.06E-07	-6.29035	S100a1
2871	M27207mF	0.000505	1.10E-07	6.280866	Col1a1
2871	rc_H33491	0.000505	1.10E-07	6.279312	Ebp
2871	U83880UTI	0.000505	1.13E-07	6.271728	Gpd2
2871	rc_AI0138€	0.000505	1.36E-07	6.217925	Hibadh
2871	M73808mF	0.000505	1.45E-07	-6.20015	Phkg2
2871	rc_AA8938	0.000505	1.45E-07	-6.19969	
2871	D25224_at	0.000505	1.47E-07	-6.19567	Rpsa
2871	D89655_at	0.000505	1.50E-07	-6.19034	Scarb1
2871	D49653_s_	0.000505	1.54E-07	6.181884	Lep
2871	rc_AA8922	0.000505	1.55E-07	-6.18132	Mgst3
2871	AB017188_	0.000532	1.69E-07	-6.15534	Psmd4
2871	rc_H33461	0.000565	1.90E-07	6.12206	Oxr1
2871	rc_AA9579	0.000565	1.96E-07	6.113201	
2871	L14323_at	0.000565	1.99E-07	6.107677	Plcb1
2871	X62671cds	0.000585	2.14E-07	-6.08784	Fau
2871	rc_AI00881	0.000746	2.89E-07	6.000456	Cycs
2871	AF055884_	0.000746	2.92E-07	-5.99676	Deaf1
2871	AF100421_	0.000746	3.01E-07	-5.9885	Mtdh
2871	D00189_at	0.000746	3.07E-07	-5.98238	Atp1a3
2871	rc_AA8497	0.000795	3.37E-07	-5.95606	Psmb1
2871	D10262_at	0.000833	3.63E-07	-5.93449	Chka
2871	U75405UTI	0.000834	3.78E-07	5.922158	Col1a1
2871	rc_AA8927	0.000834	3.82E-07	-5.91896	LOC301124
2871	AF007107_	0.000902	4.24E-07	-5.88873	Cyb5a
96	33387_at	0.000668	6.45E-09	-18.5397	GAS7
96	35817_at	0.001521	3.51E-08	-15.4941	MBP
96	32243_g_a	0.001521	5.49E-08	-14.7724	CRYAB
96	40567_at	0.001521	6.95E-08	-14.4032	TUBA1A
96	34407_at	0.001649	1.04E-07	13.79605	RARRES2
96	33917_at	0.001649	1.12E-07	-13.6905	EPB41L1

96	39178_at	0.001521	7.35E-08	-16.3926	RTN1
96	32623_at	0.002027	1.70E-07	-13.085	GABBR1
96	40422_at	0.002027	1.76E-07	13.03089	IGFBP2
96	40607_at	0.002433	2.41E-07	-12.5972	DPYSL2
96	37406_at	0.002433	2.59E-07	-12.5009	MAPRE2
96	33835_at	0.002841	3.30E-07	-12.176	TSPYL4
96	32260_at	0.003074	4.16E-07	-11.8707	PEA15
96	35778_at	0.003112	4.60E-07	-11.7397	KIF5C
96	32076_at	0.003112	5.43E-07	-11.5299	RCAN2
96	36536_at	0.003112	5.81E-07	-11.4446	SCHIP1
96	38044_at	0.003112	6.32E-07	-11.34	FAM107A
96	39385_at	0.003112	5.85E-07	12.81128	ANPEP
96	38408_at	0.003403	7.48E-07	-11.1312	TSPAN7
96	691_g_at	0.003403	7.57E-07	11.11716	P4HB
96	36939_at	0.002894	3.64E-07	-15.814	GPM6A
96	35742_at	0.004005	9.29E-07	-10.868	C16orf45
96	39760_at	0.004126	1.08E-06	-10.6929	QKI
96	36152_at	0.004126	1.08E-06	-10.6922	GDI1
96	41416_at	0.004205	1.14E-06	11.82358	FGL1
96	36666_at	0.004725	1.32E-06	10.44976	P4HB
96	32254_at	0.005188	1.61E-06	-10.225	VAMP2
96	38800_at	0.005188	1.65E-06	-10.1939	STMN2
96	36567_at	0.005011	1.50E-06	-11.4329	SLC17A7
96	37182_at	0.005455	1.81E-06	-10.09	SYP
96	40075_at	0.005455	1.85E-06	-10.0706	SYT1
96	39756_g_a	0.005806	2.04E-06	9.960928	XBP1
96	41288_at	0.005806	2.08E-06	-9.93809	CALM1 /CALM2 /CALM3
96	39331_at	0.005806	2.13E-06	-9.90903	TUBB2A
96	32715_at	0.005838	2.20E-06	9.87419	VAMP8
96	362_at	0.005944	2.30E-06	-9.82624	PRKCZ
96	38630_at	0.005011	1.47E-06	-13.1177	LASS6
96	35174_i_at	0.006537	2.59E-06	-9.69505	EEF1A2
96	35599_at	0.006547	2.68E-06	9.65951	GNMT
96	33942_s_a	0.006547	2.86E-06	-9.58607	STXBP1
96	38406_f_a	0.006547	2.88E-06	-9.57914	PTGDS
96	296_at	0.006547	2.90E-06	-9.57348	TUBB2A
96	38810_at	0.006547	2.91E-06	-9.56812	HDAC5
96	36159_s_a	0.006547	2.97E-06	-9.54558	PRNP
96	40193_at	0.007668	3.66E-06	-9.32462	ENO2
96	40653_at	0.007668	3.67E-06	-9.3209	RGS7
96	38797_at	0.007668	3.71E-06	9.311241	SLC39A14
96	37921_at	0.008094	4.11E-06	-9.20391	NPTX1
96	40825_at	0.008094	4.12E-06	-9.20013	MAPRE3
96	37365_at	0.008094	4.15E-06	-9.19343	DNAJB2
96	34767_at	0.008272	4.32E-06	-9.1514	MOAP1
96	39755_at	0.008406	4.83E-06	9.03459	XBP1
96	32607_at	0.008406	4.85E-06	-9.03071	BASP1

96	38855_s_at	0.008406	4.86E-06	-9.0284	OLFM1
96	925_at	0.008406	4.88E-06	9.02584	IFI30
96	40862_i_at	0.008812	5.20E-06	-8.96057	CKB
96	41629_at	0.008406	4.70E-06	11.20857	TM4SF4
96	40568_at	0.008994	5.39E-06	-8.9231	ATP6V1B2
96	39175_at	0.003112	5.53E-07	-18.2475	PFKP
96	40753_at	0.009343	5.69E-06	-8.86819	SNCB
96	32138_at	0.009345	5.78E-06	-8.85208	DNM1
96	35670_at	0.003112	6.24E-07	-17.9176	ATP1A3
96	1305_s_at	0.008406	4.61E-06	11.2392	CYP4F2 /// CYP4F3
96	40165_at	0.00971	6.10E-06	-8.7977	TSPYL2
4692	920252	0.000362	1.72E-09	-44.1824	Mest
4692	434767	0.008825	1.18E-07	23.38013	
4692	395082	0.008825	2.08E-07	21.44224	
4692	932079	0.008825	3.01E-07	20.27519	Ucp1
4692	552459	0.008825	3.21E-07	27.06012	Csn3
4692	372851	0.008825	3.58E-07	19.74737	Apol7c
4692	926942	0.008825	3.99E-07	-19.4269	Lipf
4692	884776	0.008825	4.02E-07	25.99746	Klk1
4692	498083	0.008825	4.36E-07	19.16774	Cd79b
4692	453230	0.008825	4.40E-07	19.14105	Cd3d
4692	520081	0.008825	5.21E-07	18.65379	Cd19
4692	568122	0.008825	6.40E-07	-18.0779	Bpifb6
4692	913882	0.008825	6.67E-07	17.96527	Fcrl1
4692	523646	0.008825	7.27E-07	17.72702	
4692	721976	0.008825	8.36E-07	17.35327	
4692	717215	0.008825	8.68E-07	-17.2555	Sfrp5
4692	517377	0.008825	8.95E-07	17.17593	Btla
4692	925254	0.008825	9.00E-07	17.1615	Cd8b1
4692	928212	0.008825	9.88E-07	16.9184	
4692	918751	0.008825	9.90E-07	16.9131	Siglech
4692	654956	0.008825	1.03E-06	16.8088	
4692	517553	0.008825	1.03E-06	-16.8024	Cyp8b1
4692	387643	0.008825	1.11E-06	16.61592	Tbc1d10c
4692	587497	0.008825	1.12E-06	16.59005	
4692	301218	0.008825	1.16E-06	16.51516	
4692	421942	0.008825	1.16E-06	16.50258	
4692	573645	0.008825	1.20E-06	16.42979	Gimap3
4692	801472	0.008825	1.26E-06	16.30018	Ccr6
4692	880995	0.008825	1.27E-06	16.28084	Cd72
4692	819976	0.008825	1.30E-06	16.22695	
4692	797351	0.008825	1.32E-06	16.18913	
4692	607840	0.008825	1.38E-06	-16.0714	Lbp
4692	508185	0.008825	1.40E-06	16.03654	Ccl19
4692	834755	0.008825	1.43E-06	15.99413	Pyhin1
4692	876665	0.009416	1.57E-06	15.76589	Ccr7
4697	703036	0.0111	5.91E-07	-14.6746	Gh

4697	414508	0.7308	7.80E-05	-8.41568	Cga
4697	922742	0.737	1.18E-04	-7.08172	Prl
4697	351994	0.8453	6.30E-04	10.40948	
4697	576185	0.8453	6.59E-04	10.28535	Wasf2
4697	359219	0.8453	7.44E-04	5.794825	
4697	578302	0.8453	7.76E-04	9.836459	Prss22
4697	836606	0.8453	8.05E-04	15.86033	
4697	925379	0.8453	8.21E-04	9.687803	Akr1c12///Akr1c13
4697	731647	0.8453	8.59E-04	15.49426	
4697	481010	0.8453	9.52E-04	9.304993	Tm4sf4
4697	503258	0.8453	1.10E-03	14.1507	
4697	740245	0.8453	1.11E-03	14.13459	Olfr715
4697	394882	0.8453	1.15E-03	13.92921	Arap3
4697	642951	0.8453	1.21E-03	13.69892	
4697	433383	0.8453	1.26E-03	8.623701	
4697	496299	0.8453	1.34E-03	8.468667	
4697	455292	0.8453	1.43E-03	12.89714	
4697	345648	0.8453	1.43E-03	12.88002	Plin4
4697	628023	0.8453	1.48E-03	12.70949	Ceacam12
4697	722499	0.8453	1.60E-03	12.37138	
4697	514577	0.8453	1.72E-03	12.04201	
4697	889516	0.8453	1.90E-03	7.691509	
4697	802117	0.8453	2.01E-03	11.39398	
4697	423363	0.8453	2.16E-03	11.08435	Olfr1388
4697	726960	0.8453	2.20E-03	7.384923	Hoxb8///Hoxb7
4697	537148	0.8453	2.25E-03	7.336838	
4697	919710	0.8453	2.27E-03	7.318424	Krtcap3
4697	774769	0.8453	2.33E-03	-4.72408	Pitpnc1
4697	927625	0.8453	2.37E-03	10.72571	Spint1
4697	570967	0.8453	2.37E-03	7.230971	
4697	861602	0.8453	2.49E-03	10.53339	Crisp3
4697	809745	0.8453	2.58E-03	7.063346	
4697	765100	0.8453	2.60E-03	-7.04612	
4697	706854	0.8453	2.62E-03	10.33537	Add1
4697	551762	0.8453	2.72E-03	10.19112	
4697	555166	0.8453	3.04E-03	9.782792	
4697	468073	0.8453	3.11E-03	-4.21629	Ncs1
4697	391299	0.8453	3.13E-03	9.678432	
4697	431194	0.8453	3.14E-03	6.682255	Syvn1
4697	686764	0.8453	3.15E-03	9.66561	Fan1
4697	637912	0.8453	3.17E-03	9.640178	H2-Ab1
4697	643417	0.8453	3.61E-03	9.191686	
4697	431239	0.8453	3.63E-03	6.417459	Cyp1a1
4697	739801	0.8453	3.66E-03	9.145989	
4697	761765	0.8453	3.75E-03	9.065753	
4697	887284	0.8453	3.77E-03	4.666326	Neurod4
4697	579707	0.8453	3.80E-03	9.020696	Mbnl3

4697	711970	0.8453	4.07E-03	8.795212	
4697	501592	0.8453	4.10E-03	6.198191	Zbtb1
4697	742371	0.8453	4.11E-03	8.763966	
4697	743542	0.8453	4.34E-03	8.586383	
4697	904044	0.8453	4.44E-03	-4.17661	U2af2
4697	734984	0.8453	4.59E-03	-3.93035	
4697	910186	0.8453	4.70E-03	5.961607	Ly6g6d
4697	440799	0.8453	4.98E-03	5.863956	Slc2a1
4697	768318	0.8453	5.01E-03	4.077644	Usp46
4697	687305	0.8453	5.08E-03	8.104154	
4697	321657	0.8453	5.22E-03	8.020458	
4697	452741	0.8453	5.24E-03	5.77894	Nalcn
4697	875485	0.8453	5.30E-03	7.974875	
4697	381228	0.8453	5.33E-03	5.750843	
4697	602022	0.8453	5.45E-03	17.005	Zbtb3
4697	577870	0.8453	5.65E-03	5.65209	Man2a1
4697	507838	0.8453	5.73E-03	7.751222	
4697	816158	0.8453	5.92E-03	7.655486	Stard6
4697	797753	0.8453	6.05E-03	16.03346	Csprs
4697	603454	0.8453	6.10E-03	7.572942	
4697	805171	0.8453	6.15E-03	7.548369	
4697	437867	0.8453	6.24E-03	7.506692	
4697	869937	0.8453	6.27E-03	15.71386	
4697	671029	0.8453	6.30E-03	3.894729	
4697	387960	0.8453	6.43E-03	15.49212	
4697	875178	0.8453	6.53E-03	15.3674	Slco2a1
4697	529442	0.8453	6.56E-03	7.370013	
4697	918411	0.8453	6.63E-03	4.574189	Ankzf1
4697	426365	0.8453	6.80E-03	7.272155	
4697	866377	0.8453	6.81E-03	-3.64774	Rph3a
4697	444707	0.8453	6.92E-03	7.223912	Wnt10a
4697	710433	0.8453	6.94E-03	14.84083	
4697	462644	0.8453	7.11E-03	5.28696	
4697	677543	0.8453	7.23E-03	-3.60664	
4697	753535	0.8453	7.24E-03	7.103013	
4697	890829	0.8453	7.28E-03	7.087737	
4697	646322	0.8453	7.46E-03	-3.58444	
4697	357505	0.8453	7.48E-03	-7.01567	Hdlbp
4697	789627	0.8453	7.63E-03	-3.74421	Caln1
4697	742280	0.8453	7.75E-03	-3.9886	Cln3
4697	633863	0.8453	7.85E-03	6.892396	2310057N15Rik
4697	601927	0.8453	7.90E-03	6.874536	Klk7
4697	429546	0.8453	8.15E-03	13.56918	
4697	905687	0.8453	8.25E-03	6.763077	
4697	897303	0.8453	8.35E-03	13.38357	
4697	475137	0.8453	8.47E-03	13.27317	Hrasls5
4697	864285	0.8453	8.47E-03	6.696009	Tmem65

4697	504508	0.8453	8.52E-03	13.2332	Olfr657///Olfr504
4697	589372	0.8453	8.52E-03	13.22645	Dip2b
4697	461975	0.8453	8.59E-03	6.661009	
4697	313790	0.8453	8.67E-03	6.64035	
4697	924680	0.8453	8.70E-03	4.982374	Col14a1
4697	436792	0.8453	8.71E-03	3.884086	Prpf38b
4697	627556	0.8453	8.80E-03	12.99014	
4697	381663	0.8453	8.85E-03	6.588466	Oprl1
4697	921686	0.8453	8.86E-03	3.628744	Spg7
4697	738869	0.8453	8.87E-03	6.58296	Grlf1
4697	580113	0.8453	8.88E-03	4.953056	
4697	519165	0.8453	8.99E-03	6.548694	Bpifa3
2392	1427660_x	0.000387	7.60E-10	35.64727	Gm10883 /
2392	1427455_x	0.002221	1.11E-08	25.10947	Gm10883 /
2392	1452417_x	0.002221	1.31E-08	24.58184	Gm10883 /
2392	1428720_s	0.009057	8.20E-08	19.31265	2010309G21Rik
2392	1425295_a	0.009057	8.89E-08	19.10708	Ear11
2392	1425519_a	0.016287	1.92E-07	17.25857	Cd74
2392	1452463_x	0.016465	2.31E-07	16.83847	Gm10883
2392	1427329_a	0.016465	2.75E-07	16.45434	Igh-6
2392	1424305_a	0.016465	2.91E-07	16.33073	Igj
2392	1425324_x	0.016766	3.29E-07	16.06485	Igh-6
2392	1447918_x	0.017884	3.86E-07	15.72661	Igl-V1
2392	1452557_a	0.018799	4.43E-07	15.44229	
2392	1438858_x	0.021354	5.53E-07	14.99313	H2-Aa
2392	1435290_x	0.021354	6.27E-07	14.74097	H2-Aa
2392	1427351_s	0.021354	6.56E-07	14.65271	Igh-6
2392	1456693_a	0.021354	6.94E-07	-14.5424	LOC100504413
2392	1425451_s	0.021354	7.18E-07	14.47809	Chi3l3 /// Chi3l4
2392	1425151_a	0.021354	7.55E-07	14.38126	Noxo1
2392	1443783_x	0.027963	1.04E-06	13.77039	H2-Aa
2392	1422386_a	0.030002	1.18E-06	13.54716	Olfr64
2392	1452431_s	0.034451	1.44E-06	13.19171	H2-Aa
2392	1452393_a	0.034451	1.49E-06	13.12788	Akna
2392	1419684_a	0.041091	1.86E-06	12.74283	Ccl8
2392	1427756_x	0.043094	2.11E-06	12.52465	Igh-6
2392	1451721_a	0.043094	2.12E-06	12.5192	H2-Ab1
3293	X60769mR	0.0949	1.12E-06	19.73167	Cebpb
3293	X17053mR	0.2617	6.16E-06	16.47086	Ccl2
3293	X63594cds	0.3119	1.1E-05	15.424	Nfkbia
3293	rc_AA8919	0.5432	2.56E-05	13.95903	LOC100365106
3293	X96437mR	0.6704	3.94E-05	13.22917	Ier3
3293	X61381cds	0.7225	0.000051	12.80364	Ifitm3
3293	D11445exc	0.7284	6E-05	12.53801	Cxcl1
3293	U22414_at	0.7655	7.51E-05	12.17301	Ccl3
3293	M32062_a	0.7655	8.11E-05	12.05072	LOC498276///Fcgr2a
3293	rc_AA8932	0.7933	0.000104	11.65785	Plin2

3293	X57523_g_	0.7933	0.000111	11.554	Tap1
3293	L18948_at	0.7933	0.000121	11.41297	S100a9
3293	M61875_s_	0.7933	0.000144	11.13711	Cd44
3293	rc_AI16932	0.7933	0.000148	11.09675	Timp1
3293	rc_AI16932	0.7933	0.000149	11.09116	Timp1
3293	D28557_s_	0.7933	0.000158	10.99757	Ybx3
3293	M14656_at	0.7933	0.000159	10.98994	Spp1
3293	J02962_at	1	0.000244	10.32751	Lgals3
3293	M34253_g_	1	0.000276	10.14373	Irf1
3293	X13044_at	1	0.00028	10.12136	Cd74
3293	rc_AA9448	1	0.000282	10.11153	Rap1b
3293	Y07704_g_	1	0.000287	10.08245	Rsad2
3293	rc_AA8927	1	0.000295	10.03984	
3293	rc_AA8188	1	0.000318	9.93058	Srgn
3293	M24026_f_	1	0.000325	9.895678	RT1-CE12
3293	L32591mRI	1	0.000353	9.773429	Gadd45a
3293	X17053cds_	1	0.00036	9.743878	Ccl2
3293	M86389cds	1	0.000369	9.706979	Hspb1
3293	rc_AA8927	1	0.000375	9.684939	Lyz2
3293	M11794cds	1	0.0004	9.587694	Mt1a
3293	rc_AI17595	1	0.000434	9.466105	Jun
3293	rc_AA8751	1	0.000444	9.4336	
3293	M65149_at	1	0.000455	9.397779	Cebpd
3293	rc_AA8923	1	0.000461	9.375997	Tuba1c
3293	U92081mR	1	0.000476	9.329486	Pdpn
3293	M97656_s_	1	0.00049	9.288221	Sstr1
3293	J05122_at	1	0.000507	9.23654	Tspo
3293	M32062_g_	1	0.000508	9.234172	Fcgr2a
3293	rc_AI10256	1	0.000524	9.189888	Mt1a
3293	rc_AI07153	1	0.000573	9.059433	Olr1
3293	rc_AI17961	1	0.00063	8.920815	Hmox1
3293	J03627_at	1	0.00069	8.789253	S100a10
3293	AF083269_	1	0.000718	8.731736	Arpc1b
3293	M15562_g_	1	0.000721	8.726889	RT1-Da
3293	X13044_g_	1	0.000721	8.726757	Cd74
3293	L13039_s_a	1	0.000732	8.705222	Anxa2
3293	rc_AA7993	1	0.000741	8.686901	Plek
3293	rc_AI17026	1	0.000805	8.568954	B2m
3293	L14782_s_a	1	0.000864	8.467791	Lyn
3293	X14254cds_	1	0.00088	8.441683	Cd74
3293	X73371_at	1	0.000889	8.426975	Fcgr2b///Fcgr2a
3293	rc_AA8927	1	0.000944	8.340947	Rgs1
3293	U75404UTI	1	0.000946	8.339193	Akap12
3293	M24067_at	1	0.00098	8.288734	Serpine1
3293	U07619_at	1	0.000981	8.287469	F3
3293	AF030089U	1	0.001054	8.18546	Dclk1
3293	S61865_s_	1	0.001056	8.182953	Sdc1

3293	rc_AA8006	1	0.001066	8.169595	Zfp36
3293	rc_AA9443	1	0.001096	8.129784	Arf6
3293	M92074_g	1	0.001111	8.110825	Tnni3
3293	D90035_s_	1	0.001168	8.040681	Ppp3ca
3293	M94919mF	1	0.001262	7.932791	Hbb-b1
3293	rc_AA9465	1	0.001427	7.760848	Lcn2
3293	M29866_s_	1	0.001467	7.722195	C3
3293	M31018_f_	1	0.001541	7.654075	Rt1.aa///RT1-EC2
3293	rc_AA8934	1	0.00171	7.510634	Rap1b
3293	rc_AA8939	1	0.001738	7.488359	
3293	S77528cds_	1	0.001783	7.452768	Cebpb
3293	S49491_s_	1	0.001867	7.389517	Penk
3293	X05834_at	1	0.002083	7.239804	Fn1
3293	rc_AI63910	1	0.00213	7.20964	Rab33b
3293	M37394_a	1	0.002139	7.203876	Egfr
3293	Z75029_s_	1	0.002197	7.167421	Hspa1a
3293	rc_AI63940	1	0.002213	7.157482	
3293	rc_AA9570	1	0.002221	7.152805	S100a8
3293	M98820_a	1	0.002282	7.115663	Il1b
3293	X52477_at	1	0.00234	7.082029	C3
3293	S56464mR	1	0.002351	7.075365	
3293	rc_AI17645	1	0.002362	7.069138	Mt2A
3293	rc_AI17196	1	0.002389	7.053617	Anxa1
3293	D15069_s_	1	0.002392	7.052042	Adm
3293	rc_AI16975	1	0.0024	7.047259	Errfi1
3293	rc_AA9444	1	0.002654	6.911526	Cnn3
3293	D45187_s_	1	0.002701	6.887755	Ctse
3293	Z54212_at	1	0.002735	6.871302	Emp1
3293	AF033355_	1	0.002791	6.843988	Pip4k2b
3293	U77777_s_	1	0.002826	6.826891	Il18
3293	M74488cds	1	0.002858	6.811841	Camk4
3293	D00913_g_	1	0.002859	6.811454	Icam1
3293	AB022209_	1	0.002894	6.795049	Hnrnpf
3293	D38065exo	1	0.002929	6.779223	Ugt1a1
3293	M94918mF	1	0.00293	6.778662	Hbb
3293	S75960_s_	1	0.00313	6.690366	Umod
3293	rc_AA8750	1	0.003155	6.679743	
3293	M63282_a	1	0.003205	6.658381	Atf3
3293	M15562_a	1	0.003348	6.600476	RT1-Da
3293	X59864mR	1	0.003415	6.57406	H19
3293	rc_AI17897	1	0.003552	6.521808	LOC287167
3293	M63122_a	1	0.003585	6.509396	Tnfrsf1a
3293	X76489cds	1	0.003593	6.506408	Cd9
3293	U53184_at	1	0.003652	6.484937	Litaf
3293	rc_AA9986	1	0.003771	6.442253	Hspb1
3293	rc_AI14568	1	0.003777	6.440259	Slc16a1
3293	rc_AI04439	1	0.00381	6.428718	Hmgb2

3293	M86341_g	1	0.00385	6.414974	Adprh
3293	U95178_s_	1	0.003901	6.397644	Dab2
3293	AF074608r	1	0.003925	6.389413	RT1-EC2
3293	AF022083_	1	0.003965	6.376001	Gnb1
3293	AF037272_	1	0.00415	6.315847	Wfdc1
3293	M96630_at	1	0.004355	6.252423	Sec61a1
3293	U09540_g_	1	0.00437	6.247877	Cyp1b1
3293	rc_AA8751	1	0.00448	6.215062	
3293	rc_AI00883	1	0.004502	6.208825	Hmgb2
3293	rc_AI63923	1	0.004519	6.203899	Phf7
3293	rc_AI04503	1	0.0046	6.180634	Cebpd
3293	L32591mRI	1	0.004638	6.169634	Gadd45a
3293	rc_AA8943	1	0.00468	6.157907	Rbpj
3293	D86297_at	1	0.004743	6.140341	Alas2
3293	rc_AI17716	1	0.004976	6.077653	Nfe2l2
3293	rc_AA8186	1	0.005	6.071389	Hspa1b///Hspa1a
3293	J04943_at	1	0.005219	6.015668	Npm1
3293	rc_AA9458	1	0.005262	6.004887	Jun
3293	AF035963_	1	0.005277	6.001083	Havcr1
3293	M15191_s_	1	0.005405	5.969937	Tac1
3293	AJ222813_	1	0.005508	5.94541	Il18
3293	rc_AI01416	1	0.005608	5.922096	Ifrd1
3293	M58364_at	1	0.00564	5.914812	Gch1
3293	U17035_s_	1	0.005658	5.910677	Cxcl10
3293	X76489cds	1	0.005708	5.899225	Cd9
3293	rc_AI17659	1	0.00572	5.896507	Ctsl1
3293	D10693_at	1	0.005742	5.891542	Hnmt
3293	M24604_at	1	0.005746	5.890462	Pcna
3293	M23697_at	1	0.0058	5.878355	Plat
3293	M63333exo	1	0.005841	5.869368	Camk4
3293	Y12319cds	1	0.006017	5.830991	Atxn3
3293	X12355_s_	1	0.006038	5.826309	Pdia3
3293	rc_AA8936	1	0.006106	5.812006	Pxn
3293	S66184_s_	1	0.006285	5.774614	Lox
3293	U41453_at	1	0.006397	5.751833	Akap12
3293	X54617mR	1	0.006425	5.746276	Myl12a
3293	rc_AI10110	1	0.006697	5.692857	Vamp2
3293	S74141_s_	1	0.006846	5.66443	Hck
3293	AF028784c	1	0.006866	5.660831	Gfap
3293	AF028784r	1	0.00688	5.658209	Gfap
3293	K03039mR	1	0.006922	5.650292	Ptprc
3293	rc_AA8940	1	0.007125	5.61328	Capg
3293	L06040_s_	1	0.00728	5.585717	Alox15
3293	U09407_at	1	0.007351	5.573192	Hint1
3293	D26393exo	1	0.007543	5.540207	Hk2
3293	J02722cds_	1	0.007785	5.499822	Hmox1
3293	X61654_at	1	0.007799	5.497579	Cd63

3293	rc_H31550_	1	0.007914	5.478893	Cpeb1
3293	M14050_s_	1	0.007945	5.473905	Hspa5
3293	AA848563_	1	0.008185	5.435989	Hspa1b///Hspa1a
3293	rc_AI63938	1	0.008208	5.43245	LOC100360017
3293	rc_AI63914	1	0.008241	5.427229	Mdfic
3293	M26744_at	1	0.008325	5.414451	Il6
3293	X67788_at	1	0.008435	5.397765	Ezr
3293	X71127_at	1	0.008487	5.389931	C1qb
3293	J03624_at	1	0.008489	5.389645	Gal
3293	L12383_at	1	0.008506	5.387109	Arf4
3293	X71127_g_	1	0.008511	5.386329	C1qb
3293	D88666_at	1	0.008539	5.38207	Pla1a
3293	rc_AI17736	1	0.008603	5.372634	Itgb1
3293	L26268_g_	1	0.008963	5.320558	Btg1
3293	rc_AI14544	1	0.008985	5.317515	Ppp1r9a
3293	M58587_at	1	0.009036	5.31037	Il6r
3293	U36899_at	1	0.009113	5.299522	Vom1r102
3293	Y00766_at	1	0.009142	5.295542	Scn3a
3293	D88534_s_	1	0.009387	5.262047	Pnlip
3293	U27558_at	1	0.009587	5.2355	Kcnj10
3293	AF087943_	1	0.009756	5.213414	Cd14
6514	1423795_a	3.68E-13	7.22E-19	6.92E+01	Sfpq
6514	1416332_a	2.76E-10	1.09E-15	5.20E+01	Cirbp
6514	1442051_a	1.38E-09	8.14E-15	4.77E+01	Hist2h3c1
6514	1422660_a	1.46E-09	1.15E-14	4.70E+01	Rbm3
6514	1452091_a	2.33E-09	2.29E-14	4.56E+01	Rbm28
6514	1435854_a	5.53E-09	6.51E-14	4.35E+01	Opalin
6514	1427464_s	6.11E-09	8.40E-14	4.30E+01	Hspa5
6514	1425993_a	1.50E-08	2.36E-13	4.10E+01	Hsph1
6514	1424638_a	3.36E-08	5.94E-13	3.92E+01	Cdkn1a
6514	1454725_a	1.42E-07	2.79E-12	3.64E+01	Tra2a
6514	1417574_a	1.61E-07	3.47E-12	3.60E+01	Cxcl12
6514	1416749_a	1.66E-07	3.92E-12	3.58E+01	Htra1
6514	1436094_a	3.05E-07	7.79E-12	3.45E+01	Vgf
6514	1420093_s	3.13E-07	8.61E-12	3.44E+01	Hnrpdl
6514	1451566_a	3.93E-07	1.16E-11	3.38E+01	Zfp810
6514	1416354_a	8.52E-07	2.68E-11	3.24E+01	Rbmx
6514	1448654_a	9.60E-07	3.21E-11	3.21E+01	Mtch2
6514	1439630_x	1.12E-06	3.96E-11	3.17E+01	Sbsn
6514	1423796_a	1.23E-06	4.58E-11	3.15E+01	Sfpq
6514	1441075_a	1.28E-06	5.03E-11	3.13E+01	Nostrin
6514	1454014_a	1.94E-06	7.98E-11	3.05E+01	Mkks
6514	1429862_a	2.03E-06	8.78E-11	3.04E+01	Pla2g4e
6514	1428470_a	2.88E-06	1.30E-10	2.97E+01	Exoc2
6514	1426722_a	3.53E-06	1.66E-10	2.93E+01	Slc38a2
6514	1442077_a	4.83E-06	2.45E-10	2.87E+01	2310076G05Rik
6514	1448454_a	4.83E-06	2.46E-10	2.86E+01	Srsf6

6514	1417303_a	6.37E-06	3.38E-10	2.81E+01	Mvd
6514	1460645_a	8.39E-06	4.61E-10	2.76E+01	Chordc1
6514	1452318_a	9.49E-06	5.53E-10	2.73E+01	Hspa1b
6514	1451047_a	9.49E-06	5.59E-10	2.73E+01	Itm2a
6514	1417606_a	1.00E-05	6.10E-10	2.72E+01	Calr
6514	1452170_a	1.05E-05	6.61E-10	2.70E+01	Chpf2
6514	1428112_a	1.08E-05	6.99E-10	2.70E+01	Manf
6514	1435158_a	1.14E-05	7.59E-10	2.68E+01	Rbm12b1
6514	1438211_s	1.63E-05	1.12E-09	2.62E+01	Dbp
6514	1416064_a	1.63E-05	1.15E-09	2.62E+01	Hspa5
6514	1455039_a	2.09E-05	1.51E-09	2.57E+01	Sin3b
6514	1415889_a	2.21E-05	1.65E-09	2.56E+01	Hsp90b1
6514	1448930_a	2.31E-05	1.77E-09	2.55E+01	3010026O09Rik
6514	1420772_a	2.62E-05	2.06E-09	2.52E+01	Tsc22d3
6514	1454711_a	3.24E-05	2.65E-09	2.48E+01	Trio
6514	1429169_a	3.24E-05	2.71E-09	2.48E+01	Rbm3
6514	1434557_a	3.24E-05	2.74E-09	2.48E+01	Hip1
6514	1445669_a	3.26E-05	2.82E-09	2.48E+01	Spry4
6514	1424182_a	3.89E-05	3.44E-09	2.44E+01	Acat1
6514	1417706_a	3.92E-05	3.54E-09	2.44E+01	Naglu
6514	1434817_s	4.09E-05	3.78E-09	2.43E+01	Rprd2
6514	1452052_s	4.29E-05	4.05E-09	2.42E+01	Eif3j1
6514	1426100_a	4.38E-05	4.22E-09	2.41E+01	Tk2
6514	1452754_a	4.61E-05	4.52E-09	2.40E+01	Creld2
6514	1457984_a	4.71E-05	4.72E-09	2.40E+01	Crh
6514	1437223_s	4.75E-05	4.85E-09	2.39E+01	Xbp1
6514	1448484_a	4.85E-05	5.04E-09	2.38E+01	Amd1
6514	1426913_a	5.09E-05	5.40E-09	2.37E+01	Lss
6514	1434618_a	5.39E-05	5.82E-09	2.36E+01	Crebzf
6514	1455444_a	5.46E-05	6.05E-09	2.36E+01	Gabra2
6514	1448471_a	5.46E-05	6.11E-09	2.36E+01	Ctla2a
6514	1424355_a	6.64E-05	7.56E-09	2.32E+01	Sin3b
6514	1427127_x	6.90E-05	7.99E-09	2.31E+01	Hspa1b
6514	1449039_a	7.54E-05	8.89E-09	2.30E+01	Hnrpdl
6514	1448130_a	7.70E-05	9.23E-09	2.29E+01	Fdft1
6514	1427126_a	7.89E-05	9.60E-09	2.29E+01	Hspa1b
6514	1428834_a	9.83E-05	1.22E-08	2.25E+01	Dusp4
6514	1442570_a	1.02E-04	1.30E-08	2.24E+01	
6514	1418174_a	1.02E-04	1.30E-08	2.24E+01	Dbp
6514	1426380_a	1.14E-04	1.48E-08	2.22E+01	Eif4b
6514	1424434_a	1.17E-04	1.53E-08	2.22E+01	Mettl22
6514	1455003_a	1.22E-04	1.63E-08	2.21E+01	Thap1
6514	1435119_a	1.32E-04	1.79E-08	2.19E+01	Gm19439
6514	1424235_a	1.39E-04	1.90E-08	2.18E+01	Ormdl2
6514	1448663_s	1.48E-04	2.07E-08	2.17E+01	Mvd
6514	1428052_a	1.48E-04	2.09E-08	2.17E+01	Zmym1
6514	1428233_a	1.70E-04	2.45E-08	2.15E+01	Cpsf6

6514	1438251_x	1.70E-04	2.48E-08	2.14E+01	Htra1
6514	1435437_a	1.70E-04	2.50E-08	2.14E+01	Setd7
6514	1420886_a	1.85E-04	2.76E-08	2.13E+01	Xbp1
6514	1434637_x	2.04E-04	3.09E-08	2.11E+01	Sin3b
6514	1455339_a	2.34E-04	3.58E-08	2.09E+01	Fkbp15
6514	1435188_a	2.44E-04	3.83E-08	2.08E+01	Gm129
6514	1430798_x	2.44E-04	3.84E-08	2.08E+01	Mrpl15
6514	1436045_a	2.47E-04	3.92E-08	2.08E+01	Tsga10
6514	1417146_a	2.56E-04	4.12E-08	2.07E+01	Mri1
6514	1416041_a	2.68E-04	4.37E-08	2.06E+01	Sgk1
6514	1416155_a	2.69E-04	4.44E-08	2.06E+01	Hmgb3
6514	1428584_a	2.77E-04	4.62E-08	2.05E+01	Haghl
6514	1448705_a	3.03E-04	5.11E-08	2.04E+01	Zbtb22
6514	1451190_a	3.26E-04	5.56E-08	2.03E+01	Sbk1
6514	1415886_a	3.35E-04	5.84E-08	2.02E+01	Sh2d3c
6514	1424033_a	3.35E-04	5.90E-08	2.02E+01	Srsf7
6514	1423723_s	3.35E-04	5.91E-08	2.02E+01	Tardbp
6514	1416811_s	3.69E-04	6.60E-08	2.00E+01	Ctla2b///Ctla2a
6514	1438635_x	3.70E-04	6.69E-08	2.00E+01	B930041F14Rik
6514	1423151_a	4.28E-04	7.82E-08	1.98E+01	Dnajb11
6514	1424068_a	4.30E-04	7.94E-08	1.97E+01	Tcta
6514	1438851_x	4.32E-04	8.15E-08	1.97E+01	Cldn1
6514	1423946_a	4.32E-04	8.18E-08	1.97E+01	Pdlim2
6514	1424481_s	4.32E-04	8.31E-08	1.97E+01	Armcx5
6514	1423667_a	4.32E-04	8.35E-08	1.97E+01	Mat2a
6514	1418902_a	4.32E-04	8.39E-08	1.97E+01	Chid1
6514	1445342_a	4.54E-04	8.91E-08	1.96E+01	
6514	1423797_a	5.06E-04	1.00E-07	1.94E+01	Aacs
6514	1435137_s	6.34E-04	1.27E-07	1.91E+01	E430024C06Rik
6514	1422943_a	6.37E-04	1.29E-07	1.91E+01	Hspb1
6514	1435902_a	6.89E-04	1.41E-07	1.89E+01	Nudt18
6514	1456170_x	7.33E-04	1.51E-07	1.88E+01	Calr
6514	1424251_a	7.36E-04	1.53E-07	1.88E+01	Hnrpdl
6514	1447223_a	7.36E-04	1.55E-07	1.88E+01	
6514	1428202_a	7.65E-04	1.62E-07	1.87E+01	Fam3a
6514	1435286_a	7.70E-04	1.65E-07	1.87E+01	Epha5
6514	1434223_a	7.70E-04	1.66E-07	1.87E+01	Oscp1
6514	1420011_s	7.73E-04	1.68E-07	1.87E+01	Xbp1
6514	1428725_a	7.82E-04	1.72E-07	1.86E+01	Pias2
6514	1452257_a	8.14E-04	1.80E-07	1.86E+01	Bdh1
6514	1428747_a	8.80E-04	1.97E-07	1.84E+01	Trnau1ap
6514	1455047_a	8.99E-04	2.03E-07	1.84E+01	Fbxo3
6514	1452094_a	9.90E-04	2.25E-07	1.83E+01	P4ha1
6514	1436209_a	1.02E-03	2.35E-07	1.82E+01	Dnajc16
6514	1455868_a	1.05E-03	2.45E-07	1.81E+01	Tubgcp2
6514	1426441_a	1.05E-03	2.45E-07	1.81E+01	Slc11a2
6514	1416609_a	1.07E-03	2.53E-07	1.81E+01	BC004004

6514	1416591_a	1.10E-03	2.61E-07	1.81E+01	Rab34
6514	1433446_a	1.28E-03	3.06E-07	1.78E+01	Hmgcs1
6514	1420365_a	1.31E-03	3.16E-07	1.78E+01	Hnrnpa2b1
6514	1459897_a	1.36E-03	3.30E-07	1.77E+01	Sbsn
6514	1416146_a	1.39E-03	3.42E-07	1.77E+01	Hspa4
6514	1416129_a	1.49E-03	3.67E-07	1.76E+01	Errfi1
6514	1425281_a	1.49E-03	3.76E-07	1.75E+01	Tsc22d3
6514	1455007_s	1.49E-03	3.76E-07	1.75E+01	Gpt2
6514	1435105_a	1.49E-03	3.77E-07	1.75E+01	Rnf208
6514	1418382_a	1.49E-03	3.83E-07	1.75E+01	Apcdd1
6514	1424591_a	1.49E-03	3.84E-07	1.75E+01	Caap1
6514	1460240_a	1.49E-03	3.85E-07	1.75E+01	Hnrnpc
6514	1417000_a	1.55E-03	4.04E-07	1.74E+01	Abtb1
6514	1435357_a	1.63E-03	4.30E-07	1.74E+01	D4Wsu53e
6514	1435880_a	1.66E-03	4.40E-07	1.73E+01	Ankrd50
6514	1437464_a	1.82E-03	4.86E-07	1.72E+01	Spata7
6514	1423608_a	1.84E-03	4.96E-07	1.72E+01	Itm2a
6514	1448351_a	1.92E-03	5.20E-07	1.71E+01	Coro1b
6514	1416461_a	1.94E-03	5.33E-07	1.71E+01	Gm20253///Caprin1
6514	1426312_a	1.94E-03	5.34E-07	1.71E+01	Bre
6514	1418706_a	1.95E-03	5.40E-07	1.70E+01	Slc38a3
6514	1450713_a	1.95E-03	5.44E-07	1.70E+01	Cspg5
6514	1448319_a	2.13E-03	5.97E-07	1.69E+01	Akr1b3
6514	1447930_a	2.26E-03	6.40E-07	1.68E+01	Baz1a
6514	1422155_a	2.29E-03	6.51E-07	1.68E+01	Hist2h3c2
6514	1435248_a	2.38E-03	6.84E-07	1.67E+01	Btaf1
6514	1424058_a	2.38E-03	6.88E-07	1.67E+01	Prrc1
6514	1452176_a	2.67E-03	7.77E-07	1.65E+01	Nup153
6514	1428958_a	2.68E-03	7.83E-07	1.65E+01	Paqr8
6514	1423474_a	2.68E-03	7.88E-07	1.65E+01	Top1
6514	1419914_s	2.73E-03	8.10E-07	1.65E+01	Nus1
6514	1417394_a	2.83E-03	8.50E-07	1.64E+01	Klf4
6514	1424074_a	2.83E-03	8.51E-07	1.64E+01	Btf3l4
6514	1451495_a	3.08E-03	9.30E-07	1.63E+01	Wac
6514	1433890_a	3.11E-03	9.47E-07	1.63E+01	Bag6
6514	1418700_a	3.11E-03	9.53E-07	1.63E+01	Lias
6514	1440867_a	3.18E-03	9.85E-07	1.62E+01	Spry4
6514	1454645_a	3.18E-03	9.87E-07	1.62E+01	Mgrn1
6514	1435826_a	3.76E-03	1.17E-06	1.60E+01	Rad18
6514	1449121_a	3.78E-03	1.19E-06	1.60E+01	Srsf10
6514	1460672_a	3.98E-03	1.26E-06	1.59E+01	2410002F23Rik
6514	1437334_x	4.02E-03	1.28E-06	1.59E+01	Parn
6514	1435374_a	4.03E-03	1.29E-06	1.58E+01	Cdyl2
6514	1436081_a	4.11E-03	1.32E-06	1.58E+01	Zfp414
6514	1434865_a	4.19E-03	1.36E-06	1.58E+01	Exoc7
6514	1425422_a	4.24E-03	1.38E-06	1.58E+01	Parn
6514	1424461_a	4.28E-03	1.40E-06	1.57E+01	Dctn2

6514	1424285_s	4.56E-03	1.51E-06	1.56E+01	Arl6ip4
6514	1455187_a	4.83E-03	1.60E-06	1.56E+01	Zbtb40
6514	1436790_a	4.99E-03	1.66E-06	1.55E+01	Sox11
6514	1428223_a	4.99E-03	1.68E-06	1.55E+01	Mfsd2a
6514	1452661_a	5.08E-03	1.72E-06	1.55E+01	Tfrc
6514	1418408_a	5.29E-03	1.80E-06	1.54E+01	Zfand1
6514	1460243_a	5.31E-03	1.81E-06	1.54E+01	Sptlc2
6514	1438075_a	5.39E-03	1.85E-06	1.54E+01	Fem1c
6514	1437151_a	5.57E-03	1.92E-06	1.53E+01	Usp22
6514	1448497_a	5.60E-03	1.95E-06	1.53E+01	Ercc3
6514	1428755_a	5.60E-03	1.96E-06	1.53E+01	Creb1
6514	1452980_a	5.98E-03	2.10E-06	1.52E+01	2810468N07Rik
6514	1434309_a	6.35E-03	2.25E-06	1.51E+01	Fntb
6514	1420500_a	6.35E-03	2.26E-06	1.51E+01	Dnajc1
6514	1456763_a	6.38E-03	2.28E-06	1.51E+01	Mprip
6514	1452247_a	6.50E-03	2.34E-06	1.50E+01	Fxr1
6514	1428917_a	6.64E-03	2.40E-06	1.50E+01	Stx17
6514	1433806_x	6.67E-03	2.42E-06	1.50E+01	Calr
6514	1448446_a	6.95E-03	2.54E-06	1.49E+01	Deaf1
6514	1426040_a	7.14E-03	2.62E-06	1.49E+01	Odf2
6514	1449184_a	7.19E-03	2.65E-06	1.49E+01	Pglyrp1
6514	1423375_a	7.23E-03	2.68E-06	1.49E+01	Cir1
6514	1435369_a	7.38E-03	2.75E-06	1.48E+01	Fastkd5
6514	1457316_a	7.40E-03	2.78E-06	1.48E+01	Map6
6514	1426402_a	7.49E-03	2.82E-06	1.48E+01	Syncrip
6514	1433700_a	7.49E-03	2.84E-06	1.48E+01	Gskip
6514	1424369_a	7.50E-03	2.86E-06	1.48E+01	Psmf1
6514	1436843_a	7.84E-03	3.00E-06	1.47E+01	Rbm12b2
6514	1447851_x	8.05E-03	3.10E-06	1.47E+01	Atp10a
6514	1428147_a	9.00E-03	3.49E-06	1.45E+01	Coro7
6514	1455105_a	9.00E-03	3.50E-06	1.45E+01	Ptpn12
6514	1438786_a	9.29E-03	3.63E-06	1.45E+01	2610021A01Rik
6514	1450674_a	9.46E-03	3.71E-06	1.44E+01	Cdk5
8700	1382553_a	0.102	4.77E-06	6.947697	Rfx3
8700	1380567_a	0.102	8.14E-06	-6.63261	
8700	1371303_a	0.102	9.84E-06	6.522384	Tdh
8700	1397039_a	0.123	1.91E-05	6.143221	
8700	1381166_a	0.123	1.99E-05	6.122652	
8700	1392329_a	0.181	3.49E-05	5.810787	Homez
8700	1370074_a	0.202	6.31E-05	-5.48995	Baiap2
8700	1370281_a	0.202	7.31E-05	-5.41184	Fabp5
8700	1369787_a	0.202	7.67E-05	5.386206	Cckar
8700	1368312_a	0.202	9.46E-05	-5.27516	Oxt
8700	1392172_a	0.202	9.51E-05	-5.2723	Ccl9
8700	1394593_a	0.202	9.64E-05	5.265458	
8700	1369049_a	0.202	9.67E-05	-5.26355	Rere
8700	1389695_a	0.202	9.69E-05	-5.26247	Fam20c

8700	1367768_a	0.202	9.72E-05	-5.26071	Lxn
8700	1368187_a	0.205	0.000106	-5.21741	Gpnmb
8700	1376321_a	0.213	0.000116	-5.16605	Piezo1
8700	1387819_a	0.225	0.00013	-5.10729	Cela1
8700	1383500_a	0.236	0.000144	-5.05558	Rrbp1
8700	1371731_a	0.239	0.000158	5.008648	Copg2
8700	1397007_a	0.239	0.000177	4.949498	
8700	1378531_a	0.239	0.00018	4.938675	
8700	1367660_a	0.239	0.00019	-4.91203	Fabp3
8700	1371487_a	0.239	0.000202	-4.88031	Sh3bgrl3
8700	1370355_a	0.239	0.000212	-4.85489	Scd1
8700	1385126_a	0.239	0.000228	4.817838	Magi2
8700	1390918_a	0.239	0.00024	-4.79278	Grtp1
8700	1391395_a	0.239	0.00027	4.732196	Ddx50
8700	1374626_a	0.239	0.000272	-4.72797	Lrg1
8700	1368930_a	0.239	0.000277	-4.71945	Kcnn4
8700	1377599_a	0.239	0.000284	-4.70628	
8700	1368037_a	0.239	0.000289	4.698307	LOC100360507///Cbr1
8700	1386877_a	0.239	0.0003	-4.67916	Ap2s1
8700	1386879_a	0.239	0.0003	-4.67792	Lgals3
8700	1374272_a	0.239	0.000301	4.676216	LOC679383
8700	1367712_a	0.239	0.000303	-4.67334	Timp1
8700	1391591_a	0.239	0.000304	4.672076	Zfp275
8700	1387581_a	0.239	0.00031	4.661992	Rassf9
8700	1373471_a	0.239	0.000311	-4.66118	Rnf166
8700	1391948_a	0.239	0.000318	4.649661	Bcl11b
8700	1371237_a	0.239	0.000321	-4.64469	Mt1a
8700	1384703_a	0.239	0.000339	4.617291	
8700	1369560_a	0.239	0.000349	-4.60142	Gpd1
8700	1387951_a	0.239	0.000351	4.598645	Cd55
8700	1394218_s	0.239	0.000355	4.593235	Zfpm2
8700	1390270_a	0.239	0.000373	4.568986	
8700	1367932_a	0.239	0.000388	-4.5489	Hmgcs1
8700	1393915_a	0.239	0.000403	-4.52939	Lpcat3
8700	1386287_a	0.239	0.000416	-4.51293	Slain2
8700	1394636_a	0.239	0.000418	4.510662	
8700	1375316_a	0.239	0.000418	4.510495	
8700	1370902_a	0.239	0.000424	-4.50391	Akr1b8
8700	1379932_a	0.239	0.000425	4.502753	Clcn4
8700	1387967_a	0.239	0.000427	-4.50091	Spink1
8700	1390383_a	0.239	0.000428	-4.49968	Plin2
8700	1390449_a	0.239	0.000437	4.488411	
8700	1375517_a	0.239	0.000442	-4.48247	Tp53inp2
8700	1370032_a	0.239	0.000452	4.471321	Slc9a3r1
8700	1381116_a	0.239	0.000454	4.469798	
8700	1378353_a	0.239	0.000461	4.462328	Setdb1
8700	1384298_a	0.245	0.000495	-4.42591	Myo1f

8700	1387834_a	0.245	0.000508	-4.41295	Matk
8700	1367609_a	0.245	0.000525	-4.39698	Mif
8700	1385036_a	0.245	0.000544	4.379371	Sncaip
8700	1381489_a	0.245	0.000554	4.369592	
8700	1387148_a	0.245	0.000564	4.360592	Gprasp1
8700	1371816_a	0.245	0.000573	4.353332	Zcchc3
8700	1391252_a	0.245	0.000573	-4.35325	Rnf41
8700	1383768_a	0.245	0.000573	4.352682	Elavl2
8700	1369788_s	0.245	0.000576	4.349991	Jun
8700	1372590_a	0.245	0.000577	-4.34968	C1qtnf1
8700	1393812_a	0.245	0.000581	4.345754	
8700	1389251_a	0.245	0.000593	-4.33618	Nudt7
8700	1374274_a	0.245	0.000623	4.311434	
8700	1392415_a	0.245	0.000637	4.300502	
8700	1369310_a	0.245	0.000647	-4.29221	Basp1
8700	1396712_a	0.245	0.000657	4.284501	
8700	1367850_a	0.245	0.000694	-4.25765	LOC100362543
8700	1367969_a	0.245	0.000709	-4.2471	Prdx6
8700	1379650_a	0.245	0.000725	4.235921	Pdzd8
8700	1377922_a	0.245	0.00073	4.232279	Zfp507
8700	1383658_a	0.245	0.000732	-4.23129	Laptm5
8700	1386859_a	0.245	0.000745	-4.2221	Tkt
8700	1382488_a	0.245	0.000749	4.219807	Ankef1
8700	1371325_a	0.245	0.000762	-4.21111	Ctsa
8700	1376456_a	0.245	0.000768	4.207241	Nmnat2
8700	1373889_a	0.245	0.000776	-4.20197	LOC363715
8700	1376786_a	0.245	0.000781	4.198744	Neo1
8700	1367633_a	0.245	0.000782	-4.19838	Glul
8700	1370377_a	0.245	0.000785	-4.1962	Cyp2d5///Cyp2d1
8700	1397800_a	0.245	0.000791	4.192715	
8700	1368671_a	0.245	0.000798	-4.18787	Srpx
8700	1388485_a	0.245	0.000799	-4.18768	Cxcl14
8700	1395390_a	0.245	0.000804	4.184549	
8700	1367632_a	0.245	0.000812	-4.17965	Glul
8700	1382435_a	0.245	0.000831	-4.16835	Fzr1
8700	1373175_a	0.245	0.00084	-4.16264	Plbd1
8700	1391153_a	0.245	0.000846	4.159386	
8700	1392857_a	0.245	0.000856	4.153363	Zscan20
8700	1374377_a	0.245	0.00086	4.151094	Ppp1r13l
8700	1375961_a	0.245	0.000863	4.149581	Frzb
8700	1377552_a	0.245	0.000863	4.149321	Zfpm1
8700	1369931_a	0.245	0.000883	-4.13806	Pkm
8700	1383131_a	0.245	0.000892	-4.1332	Itgb2
8700	1369385_a	0.245	0.000895	4.131418	Afap1
8700	1388116_a	0.245	0.000899	-4.12888	Col1a1
8700	1391951_a	0.245	0.000905	4.125985	
8700	1380428_a	0.245	0.000906	4.125334	

8700	1398537_a	0.245	0.000914	-4.12096	Amfr
8700	1395554_a	0.245	0.000926	4.114413	
8700	1386991_a	0.245	0.000929	-4.11272	Bad
8700	1380738_a	0.245	0.000932	4.111461	
8700	1385313_a	0.245	0.000935	4.109527	Pogk
8700	1391566_a	0.245	0.000941	4.106393	
8700	1382882_x	0.245	0.000944	4.104895	
8700	1391039_a	0.245	0.000965	4.094083	
8700	1371757_s	0.245	0.000987	4.083036	LOC683313
8700	1398838_a	0.245	0.000988	-4.08233	Rab7a
8700	1371402_a	0.245	0.000993	-4.08012	Atp6v1b2
8700	1367677_a	0.245	0.000993	-4.08004	Prdx5
19185	ILMN_2717	0.00133	5.17E-08	10.58453	Pim3
19185	ILMN_1233	0.00179	1.87E-07	9.530893	Gbp2b
19185	ILMN_1250	0.00179	2.09E-07	-9.44431	B930041F14Rik
19185	ILMN_1231	0.00236	3.71E-07	9.00473	Cyp4f14
19185	ILMN_2768	0.00236	4.59E-07	-8.84474	Col6a1
19185	ILMN_2672	0.00255	6.18E-07	-8.62536	Ccdc120
19185	ILMN_2708	0.00255	7.16E-07	-8.51757	Cdkn1c
19185	ILMN_2644	0.00255	9.56E-07	8.310266	Rnase4
19185	ILMN_3133	0.00255	9.77E-07	-8.29474	Mfge8
19185	ILMN_2875	0.00255	9.90E-07	8.284847	Ang
19185	ILMN_3104	0.00345	1.55E-06	7.971238	Coq10b
19185	ILMN_2696	0.00345	1.75E-06	7.888096	Npr2
19185	ILMN_1253	0.00345	1.81E-06	7.86543	Cyp2d13
19185	ILMN_2664	0.00345	1.88E-06	7.836874	Hist1h4h
19185	ILMN_2771	0.00441	2.58E-06	-7.62404	Mfge8
19185	ILMN_3031	0.00534	3.32E-06	7.454122	Coq10b
19185	ILMN_2434	0.00651	4.31E-06	7.283505	Tmem30a
19185	ILMN_2605	0.00651	4.56E-06	-7.24607	Slc6a8
19185	ILMN_1228	0.00661	4.89E-06	7.200546	Ugt2b1
19185	ILMN_2760	0.00664	5.17E-06	7.164918	Cxcl13
19185	ILMN_1259	0.00666	5.44E-06	-7.13105	Col6a1
19185	ILMN_2716	0.00711	6.09E-06	7.059425	Hist1h2bh
19185	ILMN_1234	0.0075	6.83E-06	-6.98612	Rbm3
19185	ILMN_1258	0.0075	7.01E-06	6.969443	Wnt2
19185	ILMN_2592	0.00755	7.57E-06	6.920036	Mup4
19185	ILMN_3153	0.00755	7.63E-06	6.915013	Cyp2c67
19185	ILMN_2930	0.00756	8.21E-06	6.869003	Igfbp2
19185	ILMN_2642	0.00756	8.24E-06	-6.86641	Emp1
19185	ILMN_2975	0.00756	8.65E-06	-6.8361	Rps6ka1
19185	ILMN_2652	0.00756	8.82E-06	6.823563	Hist1h2bj
19185	ILMN_2682	0.00822	1.03E-05	6.727628	Elovl3
19185	ILMN_2622	0.00822	1.05E-05	6.713226	Apol9b
19185	ILMN_2648	0.00822	1.06E-05	-6.71123	Arrdc4
19185	ILMN_2900	0.00881	1.17E-05	-6.64998	Rorc
19185	ILMN_1253	0.00962	1.31E-05	6.577718	Nudt7

39375	1427351_s	0.0197	4.36E-07	11.13447	Ighm
39375	1441219_a	0.0382	1.69E-06	9.654656	
39375	1438108_a	0.0557	3.71E-06	-8.8757	Plekhm3
39375	1447462_a	0.0934	8.28E-06	-8.12914	D7Wsu130e
39375	1419579_a	0.1183	1.55E-05	7.579565	Slc7a12
39375	1430161_a	0.1183	1.57E-05	7.567581	Dlst
39375	1455445_a	0.1285	2.00E-05	-7.36755	Cbln3
39375	1457814_a	0.188	3.91E-05	6.82004	
39375	1459159_a	0.188	3.96E-05	6.809493	Ccdc6
39375	1432897_a	0.188	4.26E-05	6.752486	4930435N07Rik
39375	1439993_a	0.188	5.26E-05	6.588525	
39375	1424098_a	0.188	5.40E-05	-6.56769	Elovl7
39375	1433293_a	0.188	5.79E-05	6.514153	Sdha
39375	1443637_a	0.188	5.92E-05	6.497413	Slc25a42
39375	1420380_a	0.188	6.25E-05	-6.45517	Ccl2
39375	1450511_a	0.1953	6.93E-05	6.376988	Musk
39375	1443138_a	0.2152	8.27E-05	6.243604	
39375	1439679_a	0.2152	8.59E-05	6.215384	Cct3
39375	1433369_a	0.2218	1.03E-04	6.08113	8430401P03Rik
39375	1457210_a	0.2218	1.04E-04	6.075841	1700092E19Rik
39375	1436725_a	0.2218	1.08E-04	-6.04885	Arhgef39
39375	1430960_a	0.2218	1.12E-04	-6.01815	4933440J02Rik
39375	1447529_a	0.2218	1.23E-04	-5.95365	
39375	1454145_a	0.2218	1.25E-04	-5.93844	Pla2r1
39375	1419793_a	0.2218	1.29E-04	5.914907	D5Ertd615e
39375	1425567_a	0.2218	1.35E-04	-5.88295	Anxa5
39375	1445706_x	0.2218	1.36E-04	5.879134	Mettl2
39375	1458149_a	0.2218	1.38E-04	5.869155	
39375	1441202_a	0.2228	1.43E-04	-5.84074	
39375	1431608_a	0.2389	1.65E-04	5.740486	Als2
39375	1444671_a	0.2389	1.68E-04	5.725144	Rasal2
39375	1419776_a	0.2389	1.70E-04	-5.71838	
39375	1441147_a	0.2389	1.75E-04	5.698785	D3Ertd229e
39375	1441627_a	0.2425	1.83E-04	5.665401	
39375	1435271_a	0.2425	1.88E-04	5.646617	Irf3
39375	1418792_a	0.2429	1.98E-04	5.610609	Sh3gl2
39375	1444806_a	0.2429	1.99E-04	5.606312	
929	98990_at	0.00102	9.18E-08	-16.6665	Bcar1
929	96993_at	0.00197	3.55E-07	10.38824	Pax5
929	98416_at	0.00262	8.55E-07	9.543666	Traf2
929	94805_f_at	0.00262	9.47E-07	9.44898	Hist1h
929	160778_at	0.00361	1.75E-06	8.895415	Uhmk1
929	161627_r_	0.00361	1.96E-06	8.798954	Prkd2
929	99928_at	0.00363	2.62E-06	-8.54583	Map1b
929	160664_at	0.00363	2.62E-06	8.545394	Frmd8
929	160744_r_	0.00471	4.09E-06	8.711054	Ctrl
929	101508_at	0.00471	4.50E-06	-8.09441	Khdrbs1

929	103926_at	0.00471	4.76E-06	8.047659	Eif4g1
929	162484_r_	0.00471	5.37E-06	7.950162	Tmed1
929	162231_r_	0.00471	5.53E-06	7.926796	Mesdc2
929	161145_f_	0.01127	1.52E-05	-7.13852	Atp5d
929	161271_r_	0.01127	1.57E-05	7.116406	
929	101312_at	0.01127	1.63E-05	7.485894	Grin2b
929	AFFX-b-Act	0.01182	1.89E-05	6.978795	Actb
929	100947_at	0.01182	1.95E-05	6.954187	
929	103227_at	0.01182	2.03E-05	13.60691	Ugt2b34
929	93712_at	0.01239	2.24E-05	6.852711	Ccnt1
929	161705_r_	0.01376	2.66E-05	6.727575	
929	95880_s_a	0.01376	2.73E-05	7.061336	C79130
929	161366_r_	0.01799	4.00E-05	6.760082	Cdh15
929	162399_f_	0.01799	4.00E-05	-6.43598	Atxn2
929	102231_at	0.01799	4.06E-05	6.425373	Creb3l1
929	AFFX-b-Act	0.01816	4.27E-05	6.391337	Actb
929	93493_at	0.01816	4.57E-05	-6.34277	Ddx5
929	92968_at	0.01816	4.69E-05	6.324479	Arf5
929	160851_r_	0.01816	5.00E-05	6.587742	Cox8b
929	101492_at	0.01816	5.17E-05	-6.25738	Pin1
929	161149_r_	0.01816	5.24E-05	6.248215	Slc23a3
929	162479_f_	0.01816	5.39E-05	-6.22934	
929	93835_at	0.01816	5.54E-05	-6.20974	Fuca1
929	103994_at	0.01816	5.92E-05	6.163699	Ago2
929	96070_at	0.01816	5.99E-05	-6.15608	Pfn1
929	161563_r_	0.01816	6.11E-05	6.143117	
929	99397_at	0.01816	6.24E-05	6.127903	Ercc2
929	162080_f_	0.01816	6.39E-05	-6.11182	Kctd17
929	96924_at	0.01816	6.52E-05	-6.09808	Wdr82
929	160585_at	0.01816	6.56E-05	-6.09391	Rpf2
929	96020_at	0.01917	7.45E-05	-6.00831	C1qb
929	93004_r_a	0.01917	7.48E-05	6.005134	BC018242
929	161348_r_	0.01917	7.54E-05	5.999875	Pdlim1
929	99486_at	0.01917	7.72E-05	5.983583	Cenpb
929	102247_at	0.01917	7.80E-05	5.977269	Ubr1
929	103501_at	0.02052	8.53E-05	5.916817	Pura
929	92592_at	0.02144	9.11E-05	5.873163	Gpd1
929	101665_at	0.02167	9.40E-05	5.852216	Nr5a1
929	AFFX-18SRI	0.02175	9.75E-05	5.827991	
929	161678_at	0.02175	9.83E-05	5.822488	Inpp5d
929	93917_at	0.02454	1.14E-04	-5.97686	Tnfsf12
929	94804_at	0.02454	1.15E-04	5.716963	Pbx1
929	161357_r_	0.02485	1.19E-04	5.696208	Gstm2
2870	1389554_a	6.16E-06	1.84E-11	814.9337	
2870	1382205_a	6.16E-06	3.63E-11	702.7191	
2870	1390838_a	8.15E-06	7.20E-11	604.8326	
2870	1385638_a	1.54E-05	1.82E-10	493.8453	

2870	1369751_a	2.33E-05	3.42E-10	429.8427	thyrotropir	Trhr
2870	1367851_a	5.07E-05	9.73E-10	341.785	prostaglan	Ptgds
2870	1367949_a	5.07E-05	1.11E-09	331.9962	proenkeph	Penk
2870	1373957_a	5.07E-05	1.19E-09	326.7376	reelin	Reln
2870	1374503_a	7.84E-05	2.08E-09	289.2824	pre-B-cell l	Pbx3
2870	1384392_a	0.000143	4.22E-09	247.3914	cytochrom	Cyp26b1
2870	1385031_a	0.000181	5.96E-09	229.2882		
2870	1378997_a	0.000181	6.38E-09	225.8625	Eph recept	Ephb6
2870	1385143_a	0.000224	9.11E-09	208.7681	similar to D	RGD1561963
2870	1368256_a	0.000224	9.25E-09	208.0744	serine (or c	Serpini1
2870	1379902_a	0.000686	3.03E-08	159.9809		
2870	1375984_a	0.000739	4.05E-08	149.9438	zinc finger	Zfhx4
2870	1369562_a	0.000739	4.07E-08	149.8073	hippocalcir	Hpcal1
2870	1376749_a	0.000739	4.24E-08	148.4294	osteoglycir	Ogn
2870	1388170_a	0.000739	4.34E-08	147.6915	potassium	Kctd1
2870	1387131_a	0.000739	4.46E-08	146.7743	serine (or c	Serpini1
2870	1372179_a	0.000739	4.57E-08	146.0153	hippocalcir	Hpcal1
2870	1377853_a	0.00083	5.47E-08	140.278	teashirt zin	Tshz3
2870	1392416_a	0.00083	5.84E-08	138.2551		
2870	1374934_a	0.00083	5.98E-08	137.505		
2870	1391071_a	0.00083	6.11E-08	136.8604		
2870	1367555_a	0.00087	6.66E-08	134.2362	albumin	Alb
2870	1384158_a	0.000978	7.94E-08	129.0592		
2870	1370063_a	0.000978	8.29E-08	127.8468	nuclear rec	Nr2f2
2870	1368059_a	0.000978	8.64E-08	126.6558	crystallin, r	Crym
2870	1378128_a	0.000978	8.89E-08	125.8605		
2870	1374818_a	0.000978	8.98E-08	125.5749	neurexoph	Nxph3
2870	1370432_a	0.000978	9.26E-08	124.7151	POU class 3	Pou3f1
2870	1372626_a	0.000978	9.50E-08	124.0074	tumor prot	Tpd52l1
2870	1367648_a	0.001134	1.14E-07	119.1554	insulin-like	Igfbp2
2870	1395431_a	0.001136	1.17E-07	118.279		
2870	1378163_a	0.001136	1.20E-07	117.6017	gremlin 2, (	Grem2
2870	1367598_a	0.001191	1.33E-07	115.002	transthyret	Ttr
2870	1368534_a	0.001191	1.34E-07	114.8922	adrenergic,	Adra1d
2870	1376645_a	0.001191	1.37E-07	114.3073	similar to R	RGD1307396
2870	1371922_a	0.001219	1.44E-07	113.0137	Carbonic ar	Car12
2870	1369093_a	0.001219	1.54E-07	111.3583	reelin	Reln
2870	1368883_a	0.001219	1.55E-07	111.1913	nephroblas	Nov
2870	1387004_a	0.001219	1.56E-07	110.9679	neuroblast	Nbl1
2870	1368395_a	0.001219	1.58E-07	110.6681	glypican 3	Gpc3
2870	1385285_a	0.00127	1.68E-07	109.107		
2870	1387907_a	0.001524	2.11E-07	103.7376	inositol 1,4	Itpr1
2870	1367845_a	0.001524	2.11E-07	103.7178	neurofilam	Nefm
2870	1387854_a	0.001595	2.25E-07	102.1768	collagen, ty	Col1a2
2870	1389120_a	0.001877	2.79E-07	97.40126	potassium	Kcnc3
2870	1376734_a	0.001877	2.79E-07	97.39491		
2870	1388936_a	0.001877	2.82E-07	97.17368	cadherin 1:	Cdh11

2870	1398616_a	0.001932	2.96E-07	96.11827	adaptor-rel	Ap1s2
2870	1370558_a	0.001974	3.08E-07	95.24754	potassium	Kcnc2
2870	1373648_a	0.001979	3.15E-07	94.78869	similar to P	LOC681849
2870	1391146_a	0.002075	3.36E-07	93.40018	cadherin 1:	Cdh11
2870	1369999_a	0.002103	3.47E-07	92.73591	neuronatin	Nnat
2870	1367887_a	0.002123	3.56E-07	92.17622	lecithin cho	Lcat
2870	1388968_a	0.002271	3.88E-07	90.43119	metallopho	Mpped1
2870	1376909_a	0.002285	3.97E-07	89.9585	RAS-like, fa	Rasl10a
2870	1383573_a	0.002363	4.21E-07	88.74209	teashirt zin	Tshz1
2870	1395052_a	0.002363	4.29E-07	88.40693		
2870	1368478_a	0.002363	4.31E-07	88.2847	dopamine	Drd1a
2870	1373098_a	0.002494	4.72E-07	86.49136	breast carc	Bcas1
2870	1382138_a	0.002494	4.74E-07	86.42412	Notch-regu	Nrarp
2870	1367691_a	0.002494	4.77E-07	86.28532	protein kin	Prkcdbp
2870	1383895_a	0.002795	5.43E-07	83.79756	dynein ligh	Dynlrb2
2870	1376319_a	0.002853	5.63E-07	83.13116	sema doma	Sema3c
2870	1389181_a	0.002948	5.90E-07	82.23569		
2870	1383353_a	0.003036	6.24E-07	81.20057		
2870	1386962_a	0.003036	6.26E-07	81.15574	phospholip	Plcb4
2870	1392867_a	0.003146	6.58E-07	80.24702		
2870	1383010_a	0.003204	6.79E-07	79.66302		
2870	1384308_a	0.003284	7.06E-07	78.97103	Meis home	Meis1
2870	1384225_a	0.003302	7.20E-07	78.60277		
2870	1380306_a	0.003302	7.37E-07	78.18876		
2870	1368542_a	0.003302	7.39E-07	78.15268	zinc finger	Zfp423
2870	1375647_a	0.00336	7.62E-07	77.6185		
2870	1372755_a	0.003381	7.79E-07	77.21777	mal, T-cell	Mal2
2870	1379594_a	0.003381	7.86E-07	77.05737		
2870	1388718_a	0.003654	8.60E-07	75.49549	tropomodu	Tmod1
2870	1371861_a	0.004095	9.76E-07	73.35646		
2870	1383644_a	0.004304	1.05E-06	72.20802		
2870	1390317_a	0.004304	1.07E-06	71.80617	similar to R	RGD1561849
2870	1389998_a	0.004304	1.08E-06	71.6724	Nuclear rec	Nr2f2
2870	1383205_a	0.004304	1.10E-06	71.45714	dapper, an	Dact2
2870	1384869_a	0.004304	1.10E-06	71.37029	epoxide hy	Ephx4
2870	1389186_a	0.004304	1.10E-06	71.35742		
2870	1369625_a	0.004672	1.22E-06	69.70514	aquaporin	Aqp1
2870	1376362_a	0.004672	1.22E-06	69.67304	neuronal p	Nptxr
2870	1392077_a	0.004705	1.28E-06	68.97086	metallopho	Mpped2
2870	1372208_a	0.004705	1.29E-06	68.84109	protein pho	Ppp1r1b
2870	1369047_a	0.004705	1.29E-06	68.83365	sulfotransf	Sult1d1
2870	1384863_a	0.004705	1.30E-06	68.72739	copine VII	Cpne7
2870	1384112_a	0.004705	1.32E-06	68.54487	5' nucleoti	Nt5e
2870	1385598_a	0.004705	1.33E-06	68.4052		
2870	1390539_a	0.004705	1.33E-06	68.36946		
2870	1377640_a	0.004922	1.41E-06	67.5098		
2870	1370434_a	0.005064	1.47E-06	66.77124	myelin-ass	Mobp

2870	1388753_a	0.005064	1.48E-06	66.73912	sulfatase 2	Sulf2
2870	1377404_a	0.005064	1.49E-06	66.60904	stanniocalc	Stc1
2870	1383699_a	0.005087	1.51E-06	66.38669	Ras-like wit	Rit2
2870	1370312_a	0.005295	1.59E-06	65.63505	spondin 1,	Spon1
2870	1384226_a	0.005313	1.61E-06	65.43677		
2870	1394786_a	0.005373	1.66E-06	64.99919	sortilin-rela	Sorl1
2870	1368144_a	0.005373	1.66E-06	64.98253	regulator o	Rgs2
2870	1375658_a	0.005592	1.77E-06	64.07157		
2870	1390923_a	0.005592	1.78E-06	63.93196	oxysterol b	Osbpl1a
2870	1372110_a	0.005592	1.79E-06	63.84455		
2870	1368443_a	0.005592	1.81E-06	63.70159	N-terminal	Necab2
2870	1391919_a	0.005592	1.82E-06	63.64359	transcriptic	Tcerg1l
2870	1387725_a	0.005592	1.86E-06	63.35791	gulonolacto	Gulo
2870	1393478_a	0.005592	1.87E-06	63.27415		
2870	1391605_a	0.005592	1.88E-06	63.20169		
2870	1395436_a	0.005592	1.88E-06	63.19003		
2870	1390958_a	0.005875	2.01E-06	62.22248	hypothetica	LOC690333
2870	1398305_a	0.005875	2.01E-06	62.20378	synaptotag	Syt17
2870	1386922_a	0.005875	2.02E-06	62.10714	carbonic ar	Car2
2870	1368587_a	0.006179	2.15E-06	61.27284	apolipopro	Apoc1
2870	1383218_a	0.006228	2.18E-06	61.04416		
2870	1375377_a	0.006308	2.23E-06	60.74782	immunoglo	Igsf3
2870	1367768_a	0.00631	2.25E-06	60.62846	latexin	Lxn
2870	1393263_a	0.006474	2.37E-06	59.92308		
2870	1382626_a	0.006474	2.37E-06	59.90677		
2870	1374168_a	0.006474	2.38E-06	59.85329		
2870	1370959_a	0.006474	2.38E-06	59.82368	collagen, ty	Col3a1
2870	1370228_a	0.006509	2.41E-06	59.6398	transferrin	Tf
2870	1398622_a	0.006603	2.47E-06	59.33635	Cysteine ric	Crim1
2870	1381133_a	0.006681	2.52E-06	59.06852		
2870	1393084_a	0.006695	2.54E-06	58.93494	HRAS-like s	Hrasls
2870	1370410_a	0.006824	2.61E-06	58.57303	immunoglo	Igsf1
2870	1382981_a	0.007051	2.72E-06	58.03098	Abelson he	Ahi1
2870	1380696_a	0.007105	2.76E-06	57.82806	family with	Fam102b
2870	1389586_a	0.007214	2.82E-06	57.52671		
2870	1385130_a	0.007357	2.90E-06	57.16755	Fez family z	Fezf2
2870	1384254_a	0.007839	3.12E-06	56.2412	OTU domai	Otud1
2870	1382114_a	0.008156	3.27E-06	55.61551	tousled-like	Tlk1
2870	1384025_a	0.008156	3.29E-06	55.54018		
2870	1373386_a	0.008191	3.33E-06	55.39313	gap junctio	Gjb2
2870	1387811_a	0.008197	3.35E-06	55.29068	angiotensir	Agt
2870	1373416_a	0.008287	3.42E-06	55.06027	Fibronectir	Fndc3b
2870	1392613_a	0.00846	3.51E-06	54.70807		
2870	1372027_a	0.008493	3.58E-06	54.48227		
2870	1386348_a	0.008493	3.58E-06	54.48151		
2870	1383422_a	0.008505	3.61E-06	54.37706	BEN domai	Bend5
2870	1396771_a	0.008859	3.78E-06	53.78017		

2870	1370048_a	0.008946	3.85E-06	53.56326	lysophosph	Lpar1
2870	1375043_a	0.008946	3.90E-06	53.4063	FBJ osteosa	Fos
2870	1374280_a	0.008946	3.92E-06	53.33394	cerebellin 2	Cbln2
2870	1374046_a	0.008946	3.95E-06	53.2332	heparan su	Hs3st2
2870	1367563_a	0.008946	3.96E-06	53.2208	secreted pr	Sparc
2870	1374546_a	0.008946	3.99E-06	53.12704		
2870	1391032_a	0.008946	4.00E-06	53.07797	seizure rela	Sez6
2870	1398689_a	0.00905	4.08E-06	52.85588	unc-79 hon	Unc79
2870	1380222_a	0.009064	4.11E-06	52.75658		
2870	1395474_a	0.00914	4.17E-06	52.57579		
2870	1376667_a	0.009227	4.24E-06	52.38291	cytochrom	Cyp26b1
2870	1396366_a	0.009242	4.27E-06	52.2858		
2870	1373487_a	0.009467	4.42E-06	51.88572		
2870	1389937_a	0.009467	4.43E-06	51.84275		
2870	1398263_a	0.009471	4.46E-06	51.76298	spectrin re	Syne1
2870	1390828_a	0.009647	4.57E-06	51.46849	neuropepti	Npy1r
2870	1391765_a	0.009796	4.67E-06	51.21177	leucine rich	Lrrc48
2870	1376893_a	0.009951	4.78E-06	50.95356	neurensin 1	Nrsn1
2870	1370841_a	0.00999	4.82E-06	50.83516	growth hor	Gh1
